# Bulk- and single cell-RNA sequencing reveal KIF20A as a key driver of hepatocellular carcinoma progression and immune evasion

**DOI:** 10.3389/fimmu.2024.1469827

**Published:** 2024-11-01

**Authors:** Zhixiong Su, Yaqi Zhong, Yufang He, Lijie You, Fuli Xin, Lei Wang, Zhihua Liu

**Affiliations:** ^1^ Department of Radiation Oncology, Jiangxi Clinical Research Center for Cancer, Jiangxi Cancer Hospital, The Second Affiliated Hospital of Nanchang Medical College, Nanchang, Jiangxi, China; ^2^ Department of Oncology, Shengli Clinical Medical College of Fujian Medical University, Fujian Provincial Hospital, Fuzhou University Affiliated Provincial Hospital, Fuzhou, China; ^3^ Department of Hepatopancreatobiliary Surgery, Fujian Medical University Cancer Hospital, Fujian Cancer Hospital, Fuzhou, Fujian, China; ^4^ Department of Hepatopancreatobiliary Surgery, Mengchao Hepatobiliary Hospital of Fujian Medical University, Fuzhou, China

**Keywords:** hepatocellular carcinoma, kinesin family member 20a, single cell RNA sequence, t cell exhaustion, immune check-point inhibitors

## Abstract

**Introduction:**

Kinesin family member 20A (KIF20A) is essential for cell proliferation and is implicated in promoting tumor progression, but its role in hepatocellular carcinoma (HCC) remains poorly studied.

**Methods:**

Through the analysis of bulk RNA-sequencing (bulk RNA-seq) and single-cell RNA sequencing (scRNA-seq) data, the expression of KIF20A and its relationship with diagnosis, prognosis, and the immune microenvironment were examined. The association between KIF20A and the malignant progression and metastasis of HCC was confirmed through *in vitro* and *in vivo* experiments. Furthermore, patient re-staging was performed using Recursive Partitioning Analysis (RPA) to enhance clinical benefit.

**Results:**

In this study, we firstly found KIF20A was overexprerssed in HCC both by bulk RNA-seq and scRNA-seq, and then the overexpression of KIF20A significantly promoted the proliferation, invasion, and metastasis in vitro. In vivo, the overexpression of KIF20A promoted the growth and lung metastasis of HCC. Furthermore, gene set variation analysis of bulk RNA-seq and scRNA-seq revealed that KIF20A might be associated with cell cycle related signaling pathways of E2F and G2M, and overexpression of KIF20A inhibited the activity of p21 and bax, as well as shortened G2 phase. Importantly, we found that KIF20A could induce T cell exhaustion via the SPP1-CD44 axe using scRNA-seq. Additionally, KIF20A was also correlated with the expression of immune checkpoint inhibitors (ICIs), and KIF20Ahigh subgroup might be benefited from the ICIs therapy.

**Conclusion:**

KIF20A emerges as a pivotal driver of HCC progression, intricately regulating cell cycle pathways and modulating immune responses, which position KIF20A as a promising target for HCC management.

## 1 Introduction

Primary liver cancer is one of the most prevalent malignancies, with approximately 906,000 new cases diagnosed globally each year, of which about 90% are hepatocellular carcinoma (HCC) (1). Radical hepatectomy remains the most cost-effective curative strategy for HCC, but many patients miss the opportunity for surgery at the time of diagnosis (2). Despite significant advances in early detection, surgical techniques, and both local and systemic treatments, the long-term prognosis remains bleak, with a 5-year survival rate of only about 20% (3, 4). Recurrence remains the primary cause of treatment failure in resectable HCC patients, with a 5-year recurrence rate as high as 75%[4]. The unique biological behavior and complex pathological mechanisms of HCC contribute to this poor prognosis, but much about these factors remains unknown (5).

HCC treatment has entered the era of molecular targeted therapy with the introduction of sorafenib (6). Advances in genomics, proteomics, and transcriptomics have led to the development of additional targeted agents, such as apatinib and lenvatinib, with promising results (7). However, challenges persist, including unsatisfactory objective response rates, fragile treatment resistance, and the lack of robust biomarkers for predicting treatment response (3). Following the IMbrave-150 trial, current guidelines recommend atezolizumab and bevacizumab as first-line systemic treatments for advanced HCC (8).

Kinesin family member 20A (KIF20A), a protein in the kinesin-6 family, uses its ATPase hydrolysis domain to regulate microtubule bundling and protein transport during mitosis (9). KIF20A also stabilizes and anchors Rab6-positive (GTPase-driven) vesicle trafficking at the Golgi apparatus (10). Unlike other kinesins, KIF20A has an additional loop motif in its motor domain, enabling specific targeting with small molecule inhibitors (11). While KIF20A has been identified in breast and renal cell cancer signatures (12, 13), it is significantly underappreciated in HCC. Nonetheless, while precision medicine has achieved considerable success in a multitude of cancer types, its implementation in HCC remains in its nascent stages. Further exploration is warranted to uncover novel precision therapeutic strategies, encompassing critical domains such as biomarkers, molecular classifications, and the heterogeneity inherent to the tumor microenvironment (14).

In this study, we initially identified the clinical significance of KIF20A in HCC using bulk and single-cell RNA (scRNA) sequencing. We then assessed its aggressive characteristics through *in vitro* and *in vivo* studies. Furthermore, we explored the underlying mechanisms by which KIF20A regulates HCC progression, the tumor immune microenvironment (TiME), and immune responses using both bulk RNA-seq and scRNA-seq.

## 2 Materials and methods

### 2.1 Data acquisition

In this study, we re-analyzed a total of 16 public datasets, including 15 bulk RNA-seq cohorts (two of which involved patients receiving immune checkpoint inhibitors) and one single-cell RNA-seq (scRNA-seq) cohort. Additionally, hepatocellular carcinoma (HCC) tissue microarrays (TMAs) were obtained from Shanghai Outdo Biotech Co., Ltd (Shanghai, China) along with corresponding clinical-pathological data and follow-up information (Ethics No.SHYJS-CP-1707015). From these, 87 samples underwent quantitative PCR (qPCR) and 66 samples were subjected to immunohistochemistry (IHC) analysis. Detailed information on these cohorts is provided in **Supplementary Table S1**, while raw PCR data from the TMA is cataloged in **Supplementary Table S2**. To ensure patient privacy, names and medical record numbers from the TMA were replaced with new research IDs.

### 2.2 ScRNA-seq analysis

As previously reported (15), initial quality control of the scRNA-seq data was performed and is displayed in **Supplementary Figure S1**. Raw scRNA-seq data were pre-processed using CellRanger (version 4.0.0) and the Seurat (version 4.0.4) pipeline in R software (version 4.1.0, R-Foundation, Vienna, Austria). The scRNA cohort consisted of 8 normal and 10 tumor samples, encompassing a total of 63,102 cells. We retained 47,578 high-quality cells for subsequent analyses based on the following criteria: 1) genes must be expressed in at least three cells; 2) cells must express at least 50 genes; 3) mitochondrial gene expression must not exceed 5%. Batch effects were removed using the “harmony” R package, and scRNA-seq data were normalized using the “Seurat” R package. We identified the top 1,500 highly variable genes and performed principal component analysis (PCA) using the “RunPCA” function. Unsupervised cell clusters were determined using the “FindClusters” function (selecting the top 20 principal components, resolution = 1.0) and visualized via uniform manifold approximation and projection (UMAP) dimensionality reduction. Marker genes for each cluster were identified using the “FindAllMarkers” function under the criteria: |logFC| > 1.0 and adjusted P-value < 0.05. Cell clusters were annotated based on marker genes of various liver cell types from the Cellmark2.0 database (http://bio-bigdata.hrbmu.edu.cn/CellMarker/index.html) and canonical cellular markers.

### 2.3 InferCNV analysis

A raw counts matrix, annotation file, and gene/chromosome position file were prepared according to instructions on the InferCNV GitHub page (https://github.com/broadinstitute/inferCNV) (16). We calculated the somatic large-scale chromosomal CNV score for each hepatocyte using the R package inferCNV (v1.6.0), with hepatocytes from normal tissue serving as reference cells. Default parameters were applied (cutoff = 0; denoise = 0.1).

### 2.4 Cell-to-cell interaction analysis

Cell-to-cell interaction analysis was performed based on the expression of specific ligands and receptors using the R package “CellChat”. This tool quantitatively infers and analyzes intercellular communication networks from scRNA-seq data (17). By leveraging manifold learning and quantitative contrasts, CellChat classifies signaling pathways and delineates both conserved and context-specific pathways across different datasets.

### 2.5 Cellular development trajectory analysis

Monocle 2 (v2.18.0) was used to infer the cellular trajectory of hepatocytes, assuming a one-dimensional “pseudotime” to describe the high-dimensional expression values of single cells (18). Cell trajectories and positions were presented in a two-dimensional plot with a tree structure after log normalization and DDR tree dimension reduction.

### 2.6 CIBERSORTx analysis

CIBERSORTx, an analytical tool for imputing gene expression profiles and estimating the abundances of cell types in mixed cell populations, was utilized (https://cibersortx.stanford.edu/) (19). We uploaded the expression matrix file of the TCGA-HCC cohort and used the “LM22” signature file to compute the proportions of 22 immune cells.

### 2.7 Functional analysis

Gene set variation analysis (GSVA) was conducted to estimate biological functions and signaling pathways in both bulk RNA-seq and scRNA-seq data (20). The reference molecular signature was “h.all.v2023.1.Hs.symbols” (downloaded from https://www.gsea-msigdb.org/gsea/msigdb/).

### 2.8 Cell culture

Human HCC cell lines Huh7, SNNU398, SNU449, SMMC7721, SK-HEP1, C3A, and MHCC97H were purchased from the American Type Culture Collection. Huh7, SMMC7721, SK-HEP1, C3A, and MHCC97H cells were cultured in DMEM medium (Gibco, California, USA), while SNNU398 and SNU449 cells were cultured in 1640 medium (Gibco, California, USA). Both media were supplemented with 10% fetal bovine serum (FBS) (Gibco, California, USA). All cells were maintained in an incubator at 37°C with 5% CO_2_.

### 2.9 Plasmids and transfection

KIF20A overexpression (OE-KIF20A) and control lentiviral plasmids were provided by OBiO Technology (Shanghai, China) and used to transfect HCC cell lines as previously described (21).

### 2.10 Orthotopic tumor growth

Sixteen male Balb/c nude mice (SPF grade; 6 weeks old; 18-22 g) were purchased from Wu’s Animal Laboratory Center Co., Ltd (Fujian, China), and all procedures were authorized by the Nanchang University Approval for Research Involving Animals (Ethics No. MHCC-AEC-2024-02). Briefly, 5×10^5 SUN449 and SMMC7721 cells, along with their corresponding OE-KIF20A cell lines, were resuspended in Matrigel (25 μL) and injected into the liver capsule of the mice. Mice were anesthetized on day 30, and liver and lung tissues were harvested for further analysis.

### 2.11 Cell counting kit-8 assay

Approximately 1×10^4 cells per well were seeded into 96-well plates and cultured for 48 hours. After 2 hours of incubation with 10 μl of CCK8 solution (MedChemExpress, Shanghai, China), the absorbance was measured at 450 nm using a spectrophotometer (Thermo Scientific, Pennsylvania, USA).

### 2.12 EdU assay

Following the protocol of the Proliferation Kit (RiboBio, Guangdong, China), cells were seeded into 24-well plates at a density of 5×10^4/well and cultured for 24 hours. Cells were then fixed with 4% paraformaldehyde after 2 hours of incubation with 5-ethynyl-2′-deoxyuridine (EdU). EdU-positive cells were counted under an Olympus FSX100 microscope (Olympus, Tokyo, Japan) to assess cell proliferation.

### 2.13 Colony formation assay

Briefly, 500 cells per well were plated into 6-well plates and cultured for approximately 10 days. Once colonies formed, they were gently washed with PBS, fixed with formalin, and stained with 0.1% crystal violet. Stained colonies were imaged and counted using ImageJ software (version 2.0.0) to evaluate cloning efficiency.

### 2.14 Cell migration and invasion assay

As previously reported, 5×10^4 cells were seeded into the upper chamber (Corning, New York, USA) with (for invasion assay) or without (for migration assay) Basement Membrane Matrigel (MG6234, Beijing, China). DMEM with or without 10% FBS was added to the lower and upper chambers, respectively. After 24 hours, cells were fixed with 4% paraformaldehyde and stained with 0.1% crystal violet solution. A Nikon inverted microscope was used to image and count stained cells to determine migration and invasion capabilities.

### 2.15 Cell cycle detection

The cell cycle was analyzed by flow cytometry (FCM). Cells in the logarithmic growth phase were stained with propidium iodide (PI) according to the manufacturer’s protocol and detected using a flow cytometer (Accuri C6 Plus; BD Pharmingen, Shanghai, China). Data were analyzed with FlowJo-V10 software (Tree Star Inc, Oregon, USA).

### 2.16 Western blot analysis

Proteins were extracted from cells in the logarithmic growth phase and quantified using a BCA protein assay. Using ACE FuturePAGE™ Precast Gels, nitrocellulose membranes were incubated with primary antibodies against p21 (CST#2947, Massachusetts, USA), p53 (CST#2524, Massachusetts, USA), Bax (CST#41162, Massachusetts, USA), Bcl2 (CST#15071, Massachusetts, USA), and Caspase 3 (CST#9668, Massachusetts, USA) overnight. After incubation with secondary antibodies for 2 hours, membranes were imaged using the ECL system (Thermo Fisher Scientific, Massachusetts, USA).

### 2.17 Immunohistochemistry staining

Tumor and peritumoral tissues were fixed overnight in 4% paraformaldehyde, paraffin-embedded, sectioned into 4 μm slices, and stained with hematoxylin and eosin (H&E), Ki67 (CST#62148, Massachusetts, USA), and KIF20A (CST#67190, Massachusetts, USA). IHC scores were determined based on the positive rate score (negative=0, 1–25%=1, 26–50%=2, 51–75%=3, 76–100%=4), with sections scoring ≥3 points classified as “high expression”.

### 2.18 Quantitative reverse transcription PCR

Relative quantitation was performed using quantitative reverse transcription polymerase chain reaction (SuperScript IV Reverse Transcriptase 18090010, Thermo Fisher Scientific, Massachusetts, USA). KIF20A-specific primers were: forward 5′-GAAAATCAGCAACCAAAC-3′ and reverse 5′-GTAAAGCATAAAAGAGACC-3′.

### 2.19 Statistical analyses

For distributed data, comparisons were made using the Student’s t-test and the Wilcoxon test, while proportions were compared using the chi-square test. Component analysis in subgroups was performed with Fisher’s exact test, and pairwise comparisons were made using the Mann-Whitney U test. Survival differences between groups were assessed using the log-rank test, and prognostic factors were identified using Cox regression analyses. All statistical analyses were conducted using RStudio version 4.0.3, with a two-sided p-value < 0.05 considered statistically significant.

## 3 Results

### 3.1 Identification and validation of KIF20A by multi-omics

**Figure 1:**
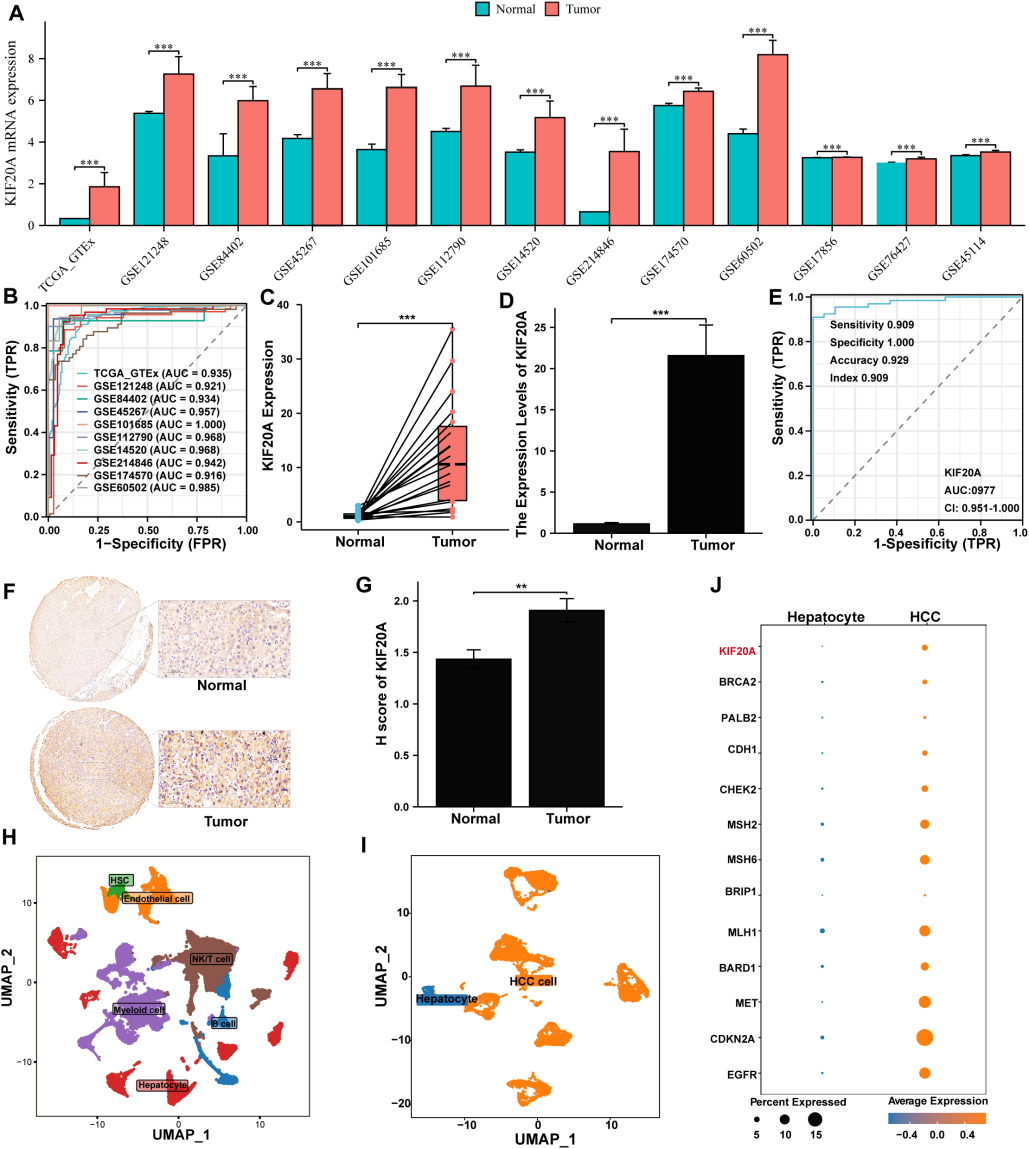
KIF20A was up-regulation in HCC cells. **(A)** The differential expression of KIF20A between normal and tumor tissues in multiply cohorts. **(B)** ROC curves of KIF20A for predicting the HCC in multiply cohorts. Differential expression of KIF20A among paired samples **(C)** and unpaired samples **(D)** in the TMA cohort by qPCR. **(E)** ROC curves of KIF20A for predicting the HCC in TMA cohort. **(F)** Typical staining histochemical results and **(G)** differential H score between normal and tumor tissue in the TMA cohort. **(H)** UMAP plot displayed cell types for all samples. **(I)** UMAP plot exhibited the distribution of hepatocyte and HCC cell. **(J)** The differential expression of KIF20A and HCC-related genes between normal hepatocytes and HCC cells. Data are presented as mean ± SD. Statistical significance was calculated by the Mann-Whitney U test. ns indicates no statistical difference, ***P < 0.001; **P <0.01.

Firstly, KIF20A RNA was found to be upregulated in tumor tissues compared to normal tissues across all datasets (all P<0.05, **Figure 1A**). This upregulation serves as a biomarker to distinguish HCC with an AUC ranging from 0.916 to 1.000 (**Figure 1B**). The overexpression of KIF20A in HCC was further confirmed through both paired and non-paired samples from TMA using qPCR (both P<0.05, **Figures 1C, D**). Additionally, the AUC of KIF20A for diagnosing HCC was notably high at 0.977 (95%CI 0.951-1.000, **Figure 1E**) in the TMA cohort. IHC analysis also confirmed KIF20A upregulation in tumor tissue of the TMA cohort (P<0.05, **Figures 1F, G**).

Furthermore, we analyzed KIF20A expression at the single-cell level. Initially, all cells underwent quality control and dimensionality reduction clustering, resulting in 44 distinct clusters (**Supplementary Figures S1A–C**). Based on the expression analysis of marker genes specific to each subgroup, the cells were categorized into NK/T cells, B cells, myeloid cells, endothelial cells, hepatocytes, and hepatic stellate cells (HSC) (**Supplementary Figure S1D**, **Figure 1H**). To further verify the upregulation of KIF20A in HCC cells, we isolated 8,497 hepatocytes from primary tumors and normal tissues, dividing them into 12 clusters (C0-C11, **Supplementary Figure S1E**). Cluster C09 was predominantly composed of normal tissue cells and used as a reference, while other clusters showed higher CNV scores (**Supplementary Figure S2**). Thus, C09 was identified as normal hepatocytes, and other clusters were identified as HCC cells (**Figure 1I**). Transcriptomics analysis revealed that HCC-related genes, such as CDKN2, MET, and MLH2, as well as KIF20A, were upregulated in the malignant cluster compared to the nonmalignant cluster (**Figure 1J**). Collectively, the upregulation of KIF20A in HCC was confirmed by both bulk RNA-seq and scRNA-seq.

### 3.2 KIF20A promotes cell proliferation, migration, and invasion in HCC Cells *in vitro*

**Figure 2:**
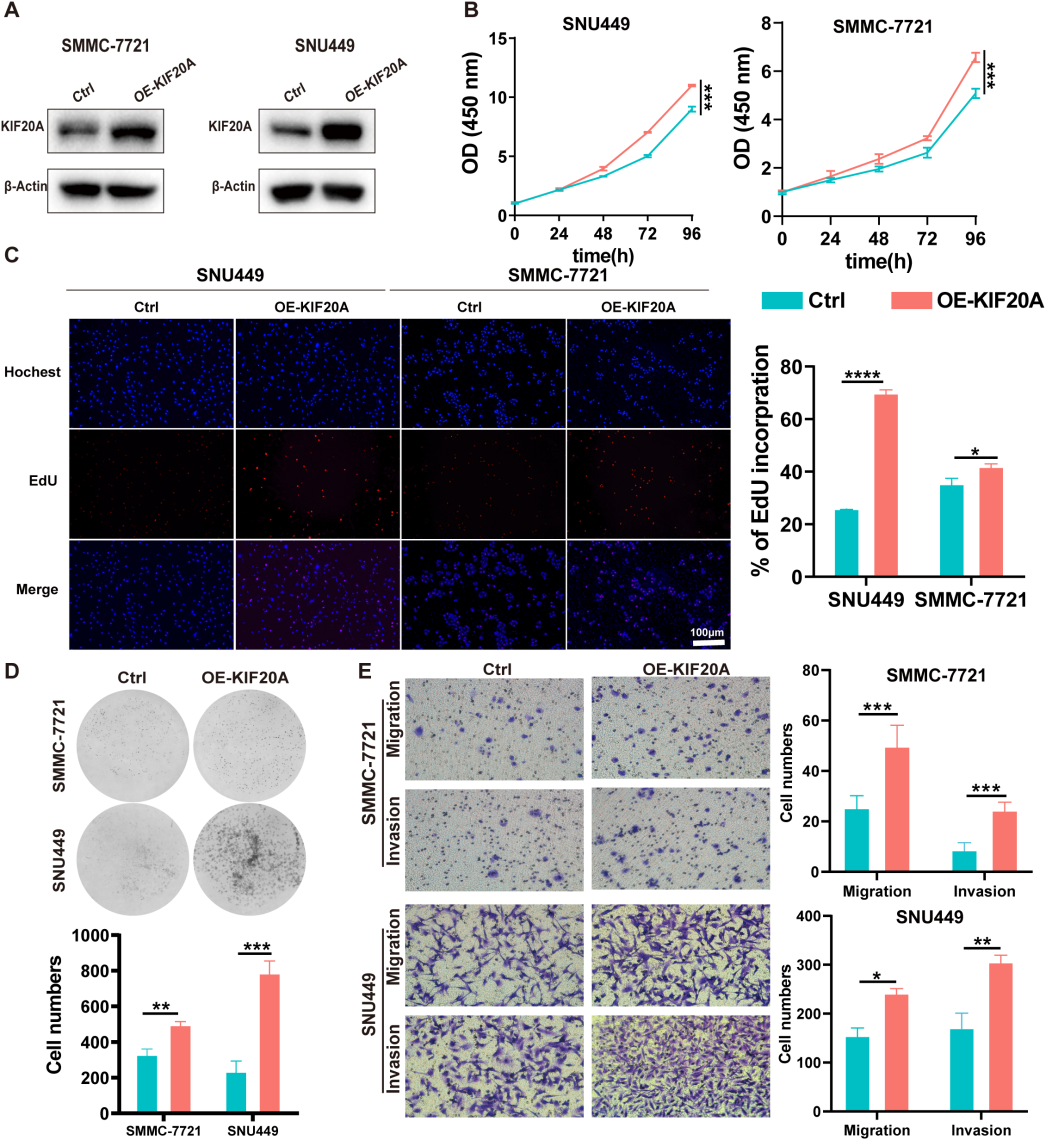
KIF20A promotes cell proliferation, migration, and invasion in HCC cells *in vitro*. **(A)** The efficiency of KIF20A overexpressed has been testified by Western blot in SMMC-7721 and SNU449. The proliferation of SMMC-7721 and SNU449 cells with KIF20A overexpressed was detected by CCK8 assay **(B)**, Edu assay **(C)** and plate clone assay **(D)**. **(E)** The cell migration and invasion of SMMC-7721 and SNU449 cells with KIF20A overexpressed was detected by transwell assays. Data are presented as mean ± SD. Statistical significance was calculated by the Mann-Whitney U test. ns indicates no statistical difference, **** P < 0.0001; ***P < 0.001; **P < 0.01; *P < 0.05.

We determined the expression of KIF20A in 7 HCC cell lines via western blot and found that KIF20A expression in SMMC-7721 and SNU449 was significantly lower compared to other HCC cell lines (**Supplementary Figure S3**). Hence, we upregulated KIF20A expression in SMMC-7721 and SNU449 cell lines using short hairpin RNAs, confirmed by western blot (OE-KIF20A-SMMC7721 and SNU449, **Figure 2A**). We then conducted CCK8, EdU, clone formation assays, invasion and migration experiments in these two OE-KIF20A HCC cell lines. Results showed that overexpression of KIF20A in SMMC7721 and SNU449 increased cell proliferation (both P<0.05, **Figure 2B**). The number of clones in OE-KIF20A cell lines was also increased, as shown by EdU (both P<0.05, **Figure 2C**) and clone formation assays (both P<0.05, **Figure 2D**). Similarly, overexpression of KIF20A resulted in increased invasion and migration in SMMC7721 and SNU449 cell lines (all P<0.05, **Figure 2E**).

### 3.3 KIF20A promotes HCC progression and metastasis *in vivo*

**Figure 3:**
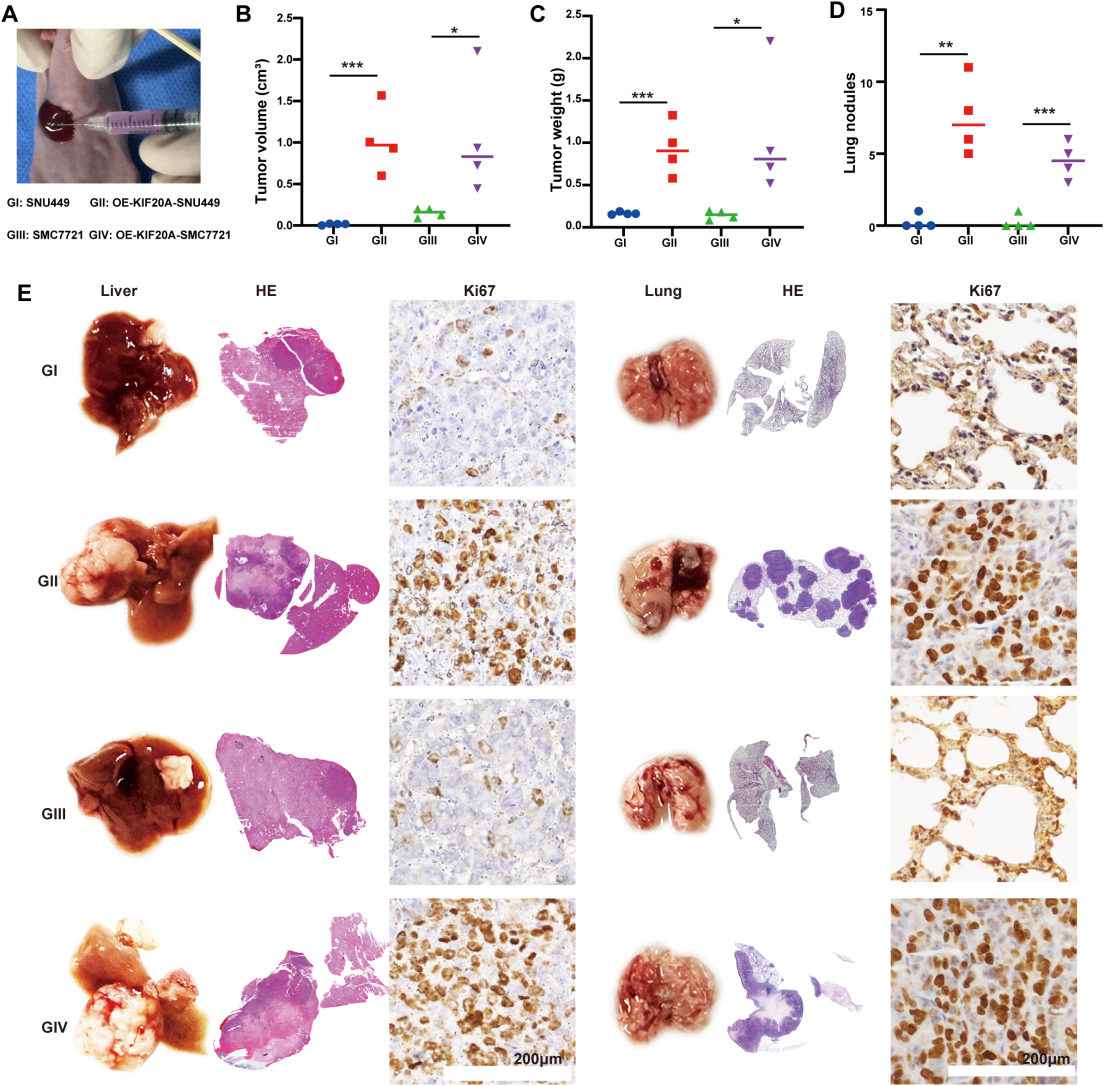
KIF20A promotes HCC progression and metastasis *in vivo*. **(A)** Establishing an *in situ* model via injecting HCC cell lines under the subcapsular of the liver. **(B)** The different of tumor volume **(B)**, weight **(C)** and lung nodules **(D)** between different groups. **(E)** Representative H&E images of the liver and lungs in each groups. Data are presented as mean ± SD. (n = 4). Statistical significance was calculated by the Mann-Whitney U test. ns indicates no statistical difference, ***P < 0.001; **P < 0.01; *P < 0.05.

We established an *in situ* model by injecting 5×10^5 HCC cells under the liver’s subcapsular region (**Figure 3A**). Four weeks later, mice were euthanized by ether inhalation. Median tumor volumes in the OE-KIF20A groups were significantly larger than in the control groups (both P<0.05, **Figure 3B**), as well as tumor weight (both P<0.05, **Figure 3C**). Additionally, the lungs of each mouse were dissected, revealing more lung nodules in the OE-KIF20A groups compared to the control groups (both P<0.05, **Figure 3D**). Representative H&E images of the liver and lungs in each group were shown in **Figure 3E**. Ki67 staining of the liver and lung indicated that KIF20A increased the proliferation and invasion of HCC cell lines (**Figure 3E**).

### 3.4 KIF20A promotes HCC progression by regulating the cell cycle

**Figure 4:**
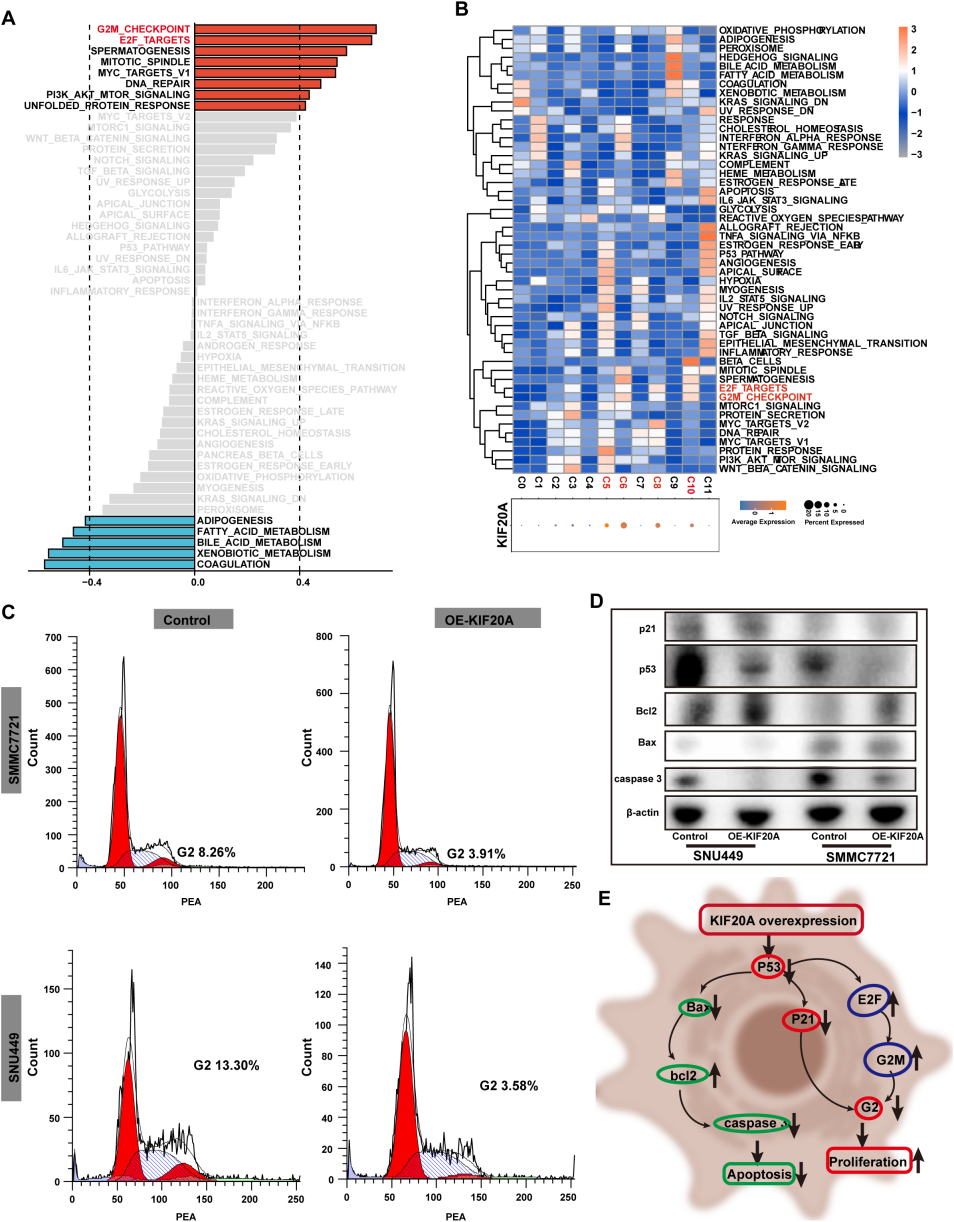
KIF20 regulated the cell cycle. **(A)** Correlation bar graphs showed the relationship between KIF20A expression and pathway activity by GSVA. **(B)** Heatmap showed the activity of hallmark pathway for hepatocytes clusters stratified by the expression of KIF20A. **(C)** FCM revealed the different proportion of cell cycle in control versus OE-KIF20A SMMC2271 and SNU449 cells. **(D)** Western blot showed the different expression of p21, p53, Bcl2, Bax and caspase between different groups in control versus OE-KIF20A SMMC-7721 and SNU449 cells. **(E)** Schematic representation of cell cycle regulation by KIF20A.

To further verify the pro-tumor effect of KIF20A, we conducted GSEA analysis using bulk RNA-seq. The results showed that cell cycle-related signaling pathways, such as the G2M checkpoint and E2F targets, were upregulated in the KIF20Ahigh subgroup (all correlation value >0.4, **Figure 4A**). Additionally, we found higher KIF20A expression in clusters C05, C06, C08, and C10 in scRNA-seq data (**Figure 4B**). Similarly, the G2M checkpoint and E2F targets pathways were upregulated in clusters with KIF20highA expression (**Figure 4B**). FCM analysis revealed a significantly shortened G2 phase in OE-KIF20A cell lines compared to control cell lines (**Figure 4C**). Proteins such as p21, p53, Bax, and caspase 3 were downregulated in OE-KIF20A cell lines, while bcl2 expression was upregulated (**Figure 4D**). These findings suggest that KIF20A may promote HCC progression by disrupting the p53-p21 or p53-E2F-G2M induced cell cycle arrest and suppressing apoptosis via blocking the p53-bax-bcl2-caspase3 pathways (**Figure 4E**), but further validation is required.

### 3.5 KIF20A overexpression induces T-cell exhausted

**Figure 5:**
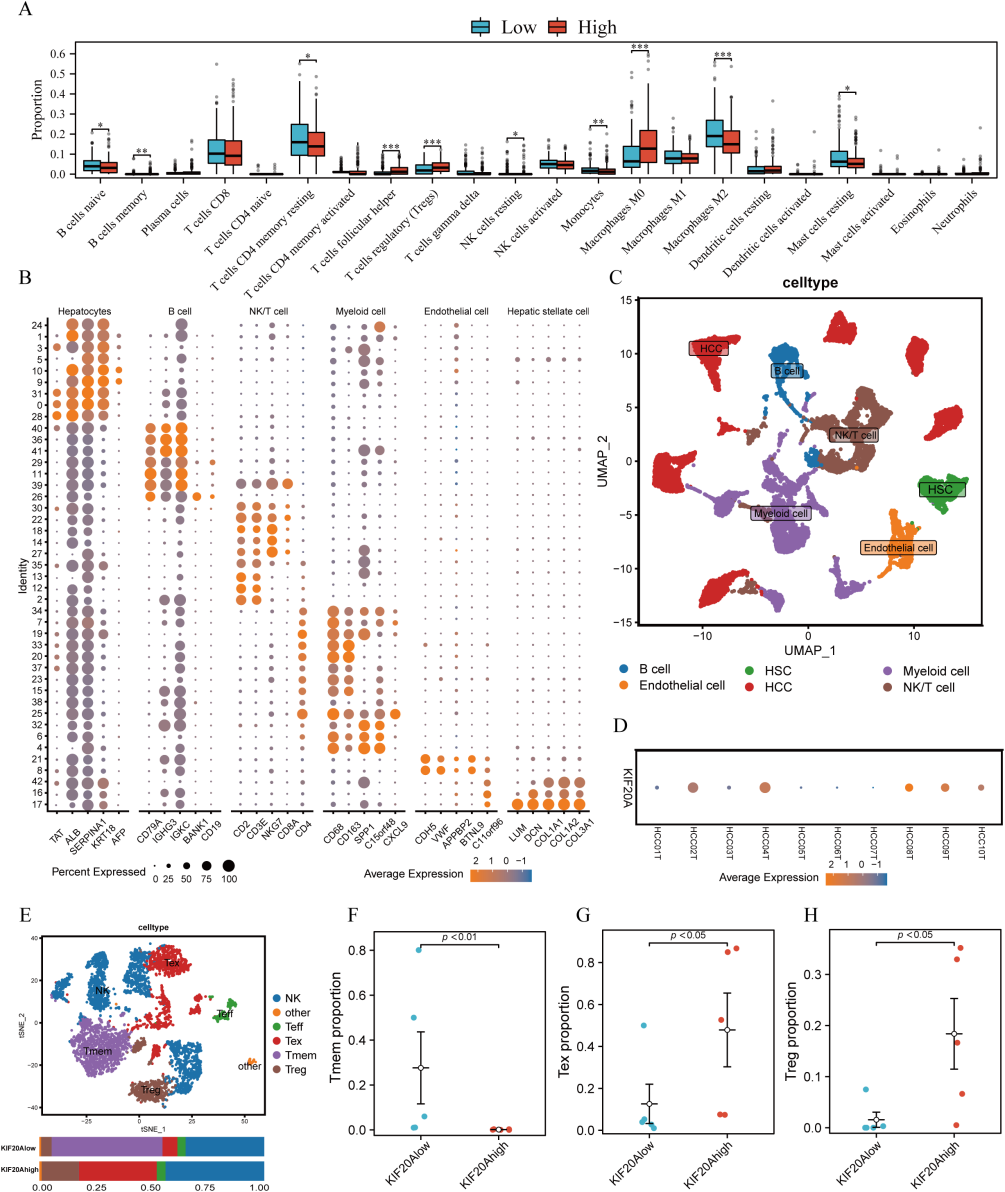
KIF20A affects immune cell infiltration in TiME. **(A)** The differential infiltration of immune cells by CIBERSORTx analysis. **(B)** The expression of corresponding markers for different cells. **(C)** UMAP plot displayed cell types for tumor samples. **(D)**The bubble plot showed the expression of KIF20A in tumor samples. **(E)** UMAP plot exhibited the cell subpopulations of NK/T cells. The different infiltrating of Tmem cells **(F)**, Tex cells **(G)** and Treg cells **(H)** between KIF20Alow and KIF20Ahigh groups. Data are presented as mean ± SD. Statistical significance was calculated by the Mann-Whitney U test. ns indicates no statistical difference, ***P < 0.001; **P < 0.01; *P < 0.05.

TiME plays a crucial role in the tumorigenesis, development, and prognosis of HCC. We evaluated 22 infiltrated immune cells between high and low KIF20A subgroups via CIBERSORTx. Results revealed that the proportions of Tregs and Macrophages M0 were significantly higher in the KIF20Ahigh subgroup compared to the KIF20Alow subgroup (both P<0.05, **Figure 5A**). Conversely, CD4 memory resting T cells (Tmem) and naive B cells were lower in the KIF20Ahigh subgroup (both P<0.05, **Figure 5A**). scRNA-seq of 10 HCC tissues confirmed this finding. After quality control (**Supplementary Figure S4**), cells were assigned to 6 distinct cell types using known marker genes: HCC, B cells, NK/T cells, myeloid cells, endothelial cells, and HSC (**Figures 5B, C**). Based on KIF20A expression, HCC tissues were categorized into KIF20Ahigh and KIF20Alow subgroups (**Figure 5D**). Given the disparities in Treg and Tmem between KIF20Alow and KIF20Ahigh observed in bulk-RNA analysis, we isolated NK/T cells for further investigation. After dimensionality reduction, clustering, and annotating, we stratified NK/T cells into NK, effective T cells (Teff), memory T cells (Tmem), Tregs, and exhausted T cells (Tex) (**Figure 5E**; **Supplementary Figures S5A, B**). Differential analysis revealed that the KIF20Alow subgroup exhibited heightened infiltration of Tmem and reduced levels of Tex and Treg infiltration, corroborating previous findings (all P<0.05, **Figures 5F–H**). Cellular development trajectory analysis indicated that Tmem differentiate into Tex over pseudotime (**Supplementary Figures S6A, B**). Collectively, these findings suggest that KIF20A overexpression may lead to T-cell exhaustion and subsequent immune escape of HCC.

### 3.6 Mechanism of KIF20A in regulating T-cell exhaustion

**Figure 6:**
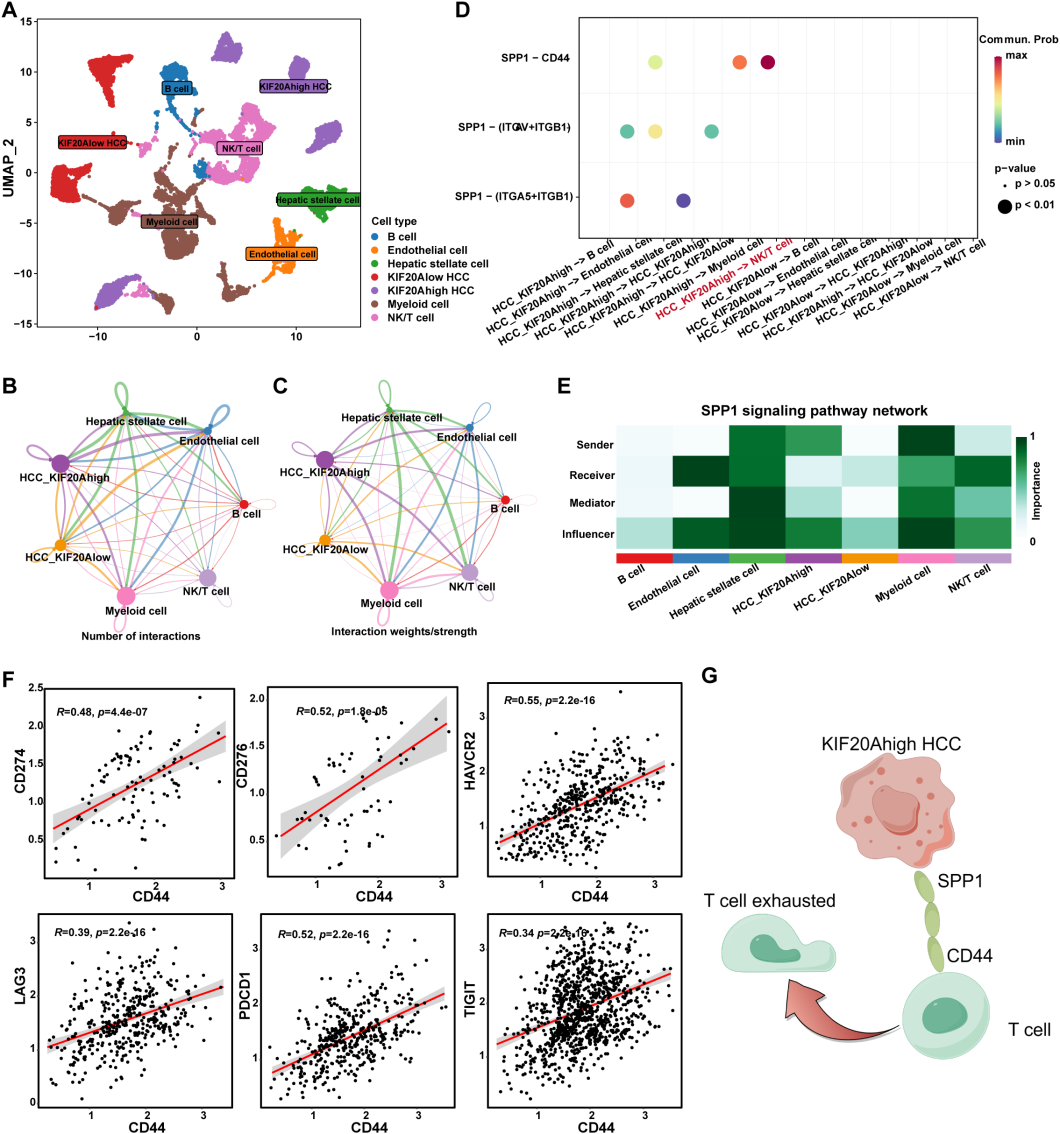
KIF20A regulating the T exhaustion. **(A)** UMAP plot showing different clusters of cells. The network plot showing the number **(B)** and intensity **(C)** of interactions between different cell populations in the TiME. **(D)** Bubble plots showing the possible ligand-receptor pairs between KIF20Ahigh/low HCC and other cell subpopulations in the TME. **(E)** The roles played by different cell populations in the tumor microenvironment in the SPP1 signaling pathway network. **(F)** The correlation of CD44 and T cell exhaustion markers in T cells, including CD274, CD276, HAVCR2, LAG3, PDCD1, and TIGIT. **(G)** Schematic representation of KIF20A regulating the T exhaustion.

To explore the mechanism by which KIF20A regulates T-cell exhaustion, we conducted a cell-to-cell communication network analysis using the R package “CellChat”. HCC cells were categorized into KIF20Ahigh and KIF20Alow groups based on KIF20A expression (**Supplementary Figure S7**), visualized in **Figure 6A**. The number and strength of each cell’s interactions were summarized in **Figures 6B, C**. Results indicated robust interactions between KIF20Ahigh HCC and NK/T cells (**Supplementary Figures S8**, **S9**). CellChat is often used to discover the potential ligand-receptor pairs between cell-to-cell. Totally, 35 significant LR pairs among the 7 cell types were detected (**Supplementary Figure S10**). Evidence shows that secreted phosphoprotein-1 (SPP1) is highly expressed in a variety of tumor types, which promotes tumor proliferation, invasion, and tumor stemness (22–24). In this study, several SPP1 ligand-receptor pairs were identified between KIF20Ahigh HCC and other cells, among which, SPP1-CD44 between KIF20Ahigh HCC and NK/T cell was the most relevant signaling pathway (**Figure 6D**). Moreover, we analyzed the role of cells in the SPP1 pathway and found that KIF20Ahigh HCC was a “sender” and NK/T cell was a “receiver” (**Figure 6E**). Further, we found that the expression of CD44 was positively correlated with CD274, LAG3, TIGIT, PDCD1, HAVCR2, and CD276 of NK/T cells, which are the classic exhaustion biomarker of T cells (all P<0.05, **Figure 6F**). Taken together, HCC might induce NK/T cell exhaustion via the KIF20A-SPP1-CD44 axis and result in an immune escape and tumor progression but the mechanism deserved further validation (**Figure 6G**).

### 3.7 Genomic alteration stratified by KIF20A expression

Genomic alterations play a crucial role in carcinogenesis, tumor progression, and treatment response (25, 26). Somatic mutations were found in 157 (90.28%) of KIF20Ahigh patients and in 156 (88.14%) of KIF20Alow patients. The top three mutations in KIF20Ahigh patients were TP53, TTN, and CTNNB1, while in KIF20Alow patients, the top mutations were CTNNB1, TTN, and ALB (**Supplementary Figures S11A, B**). The most common types of variant classifications, variant types, and single-nucleotide variant classes in both subgroups were missense mutations, single nucleotide polymorphisms, and T>G variants, respectively (**Supplementary Figures S11C, D**).

### 3.8 Immune landscape and immune response related to KIF20A

**Figure 7:**
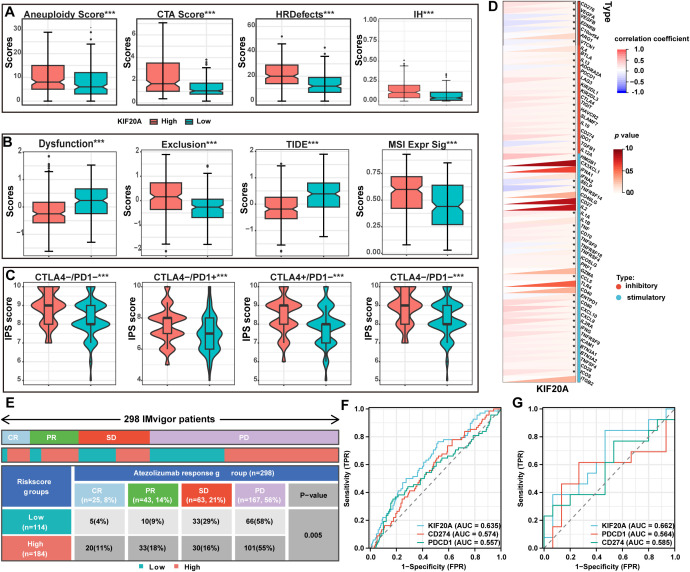
The expression of KIF20A can predict the response rate to ICIs. **(A)** The differential of immunogenicity scores between KIF20A high and low expression groups. **(B)** The differential of TIDE scores between KIF20A high and low expression groups. **(C)** The differential of IPS scores between KIF20A high and low expression groups. **(D)** The correlation of KIF20A and the genes of immune inhibitory and stimulatory. **(E)** Distribution of immune response to ICIs in different subgroups in IMvigor 210 cohort. ROC curve showing the value of KIF20A for predicting the response to ICIs in IMvigor 210 cohort **(F)** and GSE78220 **(G)**. Data are presented as mean ± SD. ns indicates no statistical difference, ***P < 0.001.

To explore the effect of KIF20A on the immune response, we examined immunogenicity, immune checkpoints, immunophenoscore (IPS) (27), and tumor immune dysfunction and exclusion (TIDE) (28) scores, Aneuploidy score, cancer testis antigens (CTA) score, homologous recombination deficiency (HRD), and intratumor heterogeneity, all stratified by KIF20A expression. The results showed that all indicators of tumor immunogenicity, including CTA score, HRD, and intratumor heterogeneity, were significantly higher in the KIF20Ahigh group than in the KIF20Alow group (P<0.001, **Figure 7A**). Next, TIDE scores were used to identify patients who would benefit from immune checkpoint inhibitors (ICIs). The TIDE and exclusion scores were significantly lower in the KIF20Ahigh group, whereas dysfunction scores and MSI expression were higher (P<0.001, **Figure 7B**). Additionally, IPS scores for CTLA-PD1+, CTLA+PD1-, and CTLA+PD1+ were significantly higher in the KIF20Ahigh group (P<0.05, **Figure 7C**). Furthermore, KIF20A was positively correlated with the expression of immune inhibitors, including CD276, CTLA4, LAG3, TIGIT, and CD274 (P<0.05, **Figure 7D**), suggesting that KIF20Ahigh patients have stronger immunogenicity and may benefit more from ICIs.

To validate these findings, we used two additional external cohorts receiving ICIs. In the IMvigor 210 cohort, 298 metastatic urothelial carcinoma patients treated with atezolizumab (a PD-1 inhibitor) showed significantly higher complete and partial response rates in the KIF20Ahigh subgroup compared to the KIF20Alow subgroup (CR: 11% vs. 4%, PR: 18% vs. 9%; P<0.05, **Figure 7E**). Additionally, the AUC for KIF20A in predicting response to PD-1 inhibitors was higher than for CD274 and PDCD1 (**Figure 7F**), a finding confirmed in another cohort of 28 melanoma patients undergoing anti-PD-1 checkpoint inhibition therapy (**Figure 7G**).

### 3.9 Novel clinical staging system based on KIF20A

**Figure 8:**
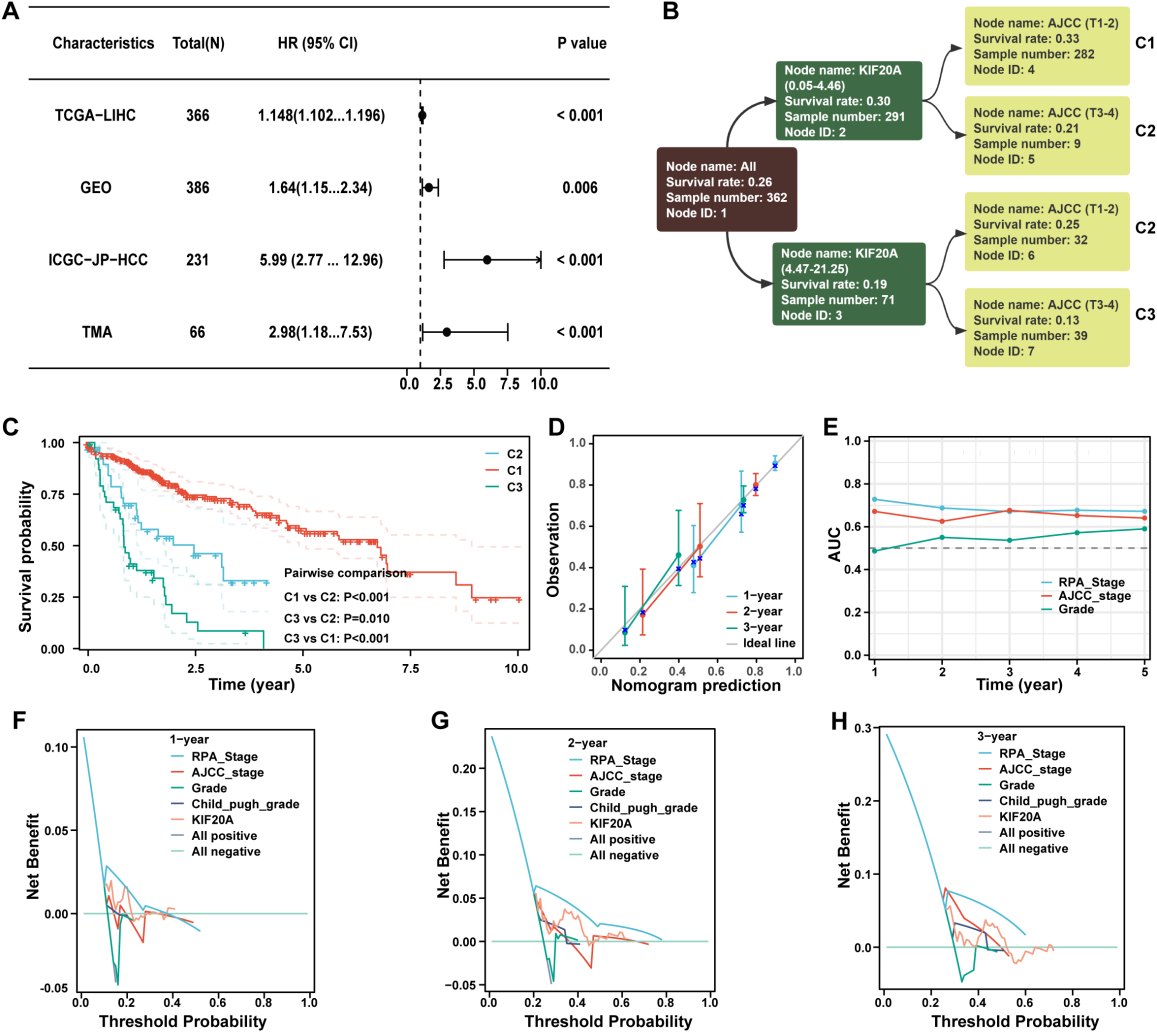
The novel stage can predict prognosis well. **(A)** The forest plot demonstrating the prognostic value of KIF20A in datasets of TCGA-LIHC, GEO, ICGC-JP-HCC and TMA. **(B)** The dendrogram of the current RPA. **(C)** Kaplan-Meier survival curve of OS between different clusters based on RPA. **(D)** Calibration curves showed the concordance between predicted and observed 1-, 2-, and 3-years survival rates. **(E)** The broken line graph showing the AUCs of RPA_stage, AJCC_stage and grade. **(F–H)** Disease curves analysis for RPA_stage, AJCC_stage, grade, child_pugh_grade and KIF20A at 1-, 2-, and 3-years to assess clinical utility in TCGA-HCC cohort.

Recursive Partitioning Analysis (RPA) is commonly used to construct prognostic re-staging models and compare their performance. KIF20A expression was identified as a robust independent risk factor for HCC in the public cohorts of TCGA-LIHC, GEO, ICGC, and TMA (P<0.05, **Figure 8A**), as well as in the AJCC-T stage (P<0.05, **Supplementary Table S3**). Using RPA analysis, we categorized 362 HCC patients into three clusters with distinct prognoses: C1 (AJCC T1-3 with KIF20A of 0.05-4.46), C2 (AJCC T3-4 with KIF20A of 0.05-4.46 and T1-2 with KIF20A of 4.47-21.25), and C3 (AJCC T3-4 with KIF20A of 4.47-21.25; P<0.05, **Figures 8B, C**). High consistency was observed between the calibration of 1-, 2-, and 3-year predicted prognosis of RPA stage vs. observed prognosis (**Figure 8D**), with improved time-dependent AUC (**Figure 8E**). Furthermore, the RPA stage demonstrated better 1-, 2-, and 3-year survival net benefits than the AJCC staging system, grade, Child-Pugh, and KIF20A alone, as shown by decision-making curves (**Figures 8F–H**).

Drug sensitivity analysis based on RPA stage showed that the IC50 of doxorubicin, mitomycin C, and gemcitabine in the C1 subgroup were significantly higher than in the C2 and C3 subgroups (P<0.05, **Supplementary Figure S12**). Conversely, the IC50 of axitinib, gefitinib, nilotinib, lapatinib, erlotinib, and sorafenib in the C1 subgroup were significantly lower than in the C2 or C3 subgroups (P<0.05, **Supplementary Figure S13**).

## 4 Discussion

HCC exhibits high heterogeneity in molecular profiles and clinical outcomes, prompting ongoing research to identify key drivers. Bulk RNA-seq is commonly employed to screen candidate drivers, but systems with substantial internal cell heterogeneity may lose valuable information on abnormal gene expression (29). Recent advances in scRNA-seq have enabled the discovery of new cell subsets and investigation of intercellular heterogeneity, although the sequencing depth of single-cell transcriptomics may not match that of bulk samples with a higher signal-to-noise ratio (30). onsequently, joint analyses of bulk RNA-seq and scRNA-seq have been attempted to elucidate uncertain relationships in disease progression.

In prior studies, activated KIF20A was found to promote HCC proliferation and was linked to poor patient prognosis (31–33). Our study revealed that KIF20A is upregulated in HCC tissues across multiple GEO cohorts using bulk RNA-seq, TMA using qPCR and IHC, and one cohort using scRNA-seq. Notably, KIF20A demonstrated excellent predictive ability, distinct from published gene prognosis-predicting signatures. Overexpression of KIF20A in two HCC cell lines (SMMC7721 and SNU449) enhanced proliferation, invasion, and metastasis *in vitro* and *in vivo*, underscoring its crucial role in HCC progression.

Cell cycle dysregulation is a hallmark of cancer, disrupting the balance of oncogenes and tumor suppressor genes, activating proliferation-related pathways, and promoting excessive cell division (34). Our study found that KIF20A is highly associated with cell cycle-related signaling pathways such as G2M and E2F, as well as apoptosis, using GSEA in both bulk RNA-seq and scRNA-seq. FCM results revealed that overexpression of KIF20A increased the proportion of the G1 phase. Furthermore, apoptosis-related proteins like p21, p53, bax, and caspase 3 were downregulated, while bcl2 was upregulated by overexpression of KIF20A. Thus, KIF20A might not only participate in cell cycle arrest via the p53-p21 or p53-E2F-G2M pathways but also suppress apoptosis via the p53-bax-bcl2-caspase3 pathway.

T cell exhaustion remains an ongoing concern in cancer research (35, 36). Different from traditional views, Rudloff et al (37) found that CD8+ T cell exhaustion could occur as soon as 6 hours after contact with the tumor. In this study, we first observed a decrease in CD8+ T cells in the KIF20Ahigh group compared to the KIF20Alow group using bulk RNA-seq, and subgroups of Tex and Treg were increased in the KIF20Ahigh group using scRNA-seq. These findings suggest that KIF20A might induce CD8+ T cell exhaustion. Moreover, while exhaustive T cells may still proliferate in human tumors (38), the proliferation mechanism remains unknown. We found that the Tmem subgroup was decreased in the KIF20Ahigh group compared to the KIF20Alow group using scRNA-seq, and cellular development trajectory analysis revealed that Tmem differentiates into Tex over pseudotime. Further, cellChat analysis suggested that the most correlated signaling pathway between KIF20Ahigh HCC and NK/T cells was SPP1-CD44. As a receptor, CD44 was also correlated with exhaustion biomarkers CD274, LAG3, TIGIT, PDCD1, HAVCR2, and CD276. Consequently, we speculated that KIF20A might promote the differentiation of Tmem into Tex via the SPP1-CD44 axis, resulting in immune escape.

Tumors can influence their TiME by releasing signaling molecules, promoting angiogenesis, and inducing immune tolerance (39). Conversely, TiME can affect the growth and development of cancer cells (40, 41). We found that immunogenicity within KIF20Ahigh HCC was higher than within KIF20Alow HCC. KIF20A correlated positively with immune checkpoint inhibitors CD274, CD276, CTLA4, and PDCD1. Additionally, KIF20Ahigh patients had lower TIDE and higher MSI scores compared to KIF20Alow patients, indicating that KIF20Ahigh patients may benefit more from ICIs, a finding confirmed by IPS analysis. These findings were validated in the IMbrave cohort of 298 patients receiving ICIs. KIF20A outperformed CTLA4 and PD-L1 in predicting response, suggesting that KIF20A could serve as an alternative biomarker in ICI decision-making.

The modified TNM staging system, incorporating KIF20A, is another highlight of this study. After confirming the independent prognostic value of KIF20A across multiple cohorts, we integrated it into the current TNM staging system using RPA. The novel staging system categorized patients into three clusters (C1, C2, and C3) with distinct prognoses. DCA analysis showed that the novel staging system offered better clinical net benefits than the current staging system alone. Additionally, drug susceptibility testing revealed significant differences in the IC50 of chemotherapy agents and targeted agents among the three clusters, indicating that the novel staging system could guide chemotherapy and targeted therapy management.

However, this study also has some limitations that can be further explored in future work. Firstly, more detailed *in vivo* animal experiment data, including liver-to-body weight ratio, can provide more information on the impact of KIF20A on tumor burden. Secondly, the impact of KIF20A on the genome can be further analyzed through CRISPR screening technology (42). Finally, multi-dimensional single-cell sequencing technology with spatial and temporal genomics can provide more reliable evidence on the impact of KIF20A on the tumor microenvironment (43).

## 5 Conclusion

In this study, we have established KIF20A as a significant player in the progression of HCC. Through comprehensive multi-omics analysis, including bulk RNA-seq and single-cell RNA-seq, we confirmed the upregulation of KIF20A in HCC tissues. Our *in vitro* and *in vivo* experiments demonstrated that KIF20A promotes HCC cell proliferation, migration, and invasion. Mechanistically, KIF20A influences key cell cycle and apoptosis pathways, contributing to tumor growth and metastasis. Furthermore, KIF20A was shown to induce T-cell exhaustion, potentially facilitating immune escape. Importantly, KIF20A’s correlation with immune checkpoint inhibitors suggests that KIF20Ahigh patients could benefit more from immune checkpoint inhibition therapies. Finally, our novel TNM staging system incorporating KIF20A expression provided superior prognostic accuracy and clinical utility, offering a valuable tool for guiding personalized treatment strategies in HCC. These findings underscore the potential of KIF20A as both a prognostic biomarker and a therapeutic target in HCC.

## Data Availability

The original contributions presented in the study are included in the article/**Supplementary Materials**, further inquiries can be directed to the corresponding authors.
