# Supplementary material for: Bulk- and single cell-RNA sequencing reveal KIF20A as a key driver of hepatocellular carcinoma progression and immune evasion
Source: Front Immunol. 2024 Nov 1;15:1469827. doi: 10.3389/fimmu.2024.1469827 (PMC11563802; doi:10.3389/fimmu.2024.1469827)
Supplement: Supplementary file 1 [file DataSheet1.docx]

**Bulk- and single cell-RNA sequencing Reveal KIF20A as a Key Driver of Hepatocellular Carcinoma Progression and Immune Evasion**

**Zhixiong Su^1,2†^,** **Yaqi Zhong^3†^, Yufang He^2†^, Lijie You^2^, Fuli Xin^4,5^, Lei Wang^1*^, Zhihua Liu^1*^**

^1^Department of Radiation Oncology, Jiangxi Clinical Research Center for Cancer, Jiangxi Cancer Hospital, The Second Affiliated Hospital of Nanchang Medical College, Nanchang, Jiangxi, China.

^2^Department of Oncology, Shengli Clinical Medical College of Fujian Medical University, Fujian Provincial Hospital, Fuzhou, 350001, China

^3^Department of Hepatopancreatobiliary Surgery, Fujian Medical University Cancer Hospital, Fujian Cancer Hospital, Fuzhou 350014, China

^4^Department of Hepatopancreatobiliary Surgery, Mengchao Hepatobiliary Hospital of Fujian Medical University, Fuzhou, 350001, China

^5^Department of Hepatopancreatobiliary Surgery, Fujian Medical University Cancer Hospital, Fujian Cancer Hospital, Fuzhou 350014, China

† contributed equally to this works

*Correspondence:

Lei Wang, Department of Radiation Oncology, Jiangxi Clinical Research Center for Cancer, Jiangxi Cancer Hospital, The Second Affiliated Hospital of Nanchang Medical College, Nanchang, Jiangxi, China. E-mail: wangleiy001@126.com

Zhihua Liu, Department of Radiation Oncology, Jiangxi Clinical Research Center for Cancer, Jiangxi Cancer Hospital, The Second Affiliated Hospital of Nanchang Medical College, Nanchang, Jiangxi, China. E-mail: lzh20130501@163.com

**Supplementary materials**

**Supplementary tables**

Supplementary table 1. Data set information used in this article.

Supplementary table 2. The raw PCR data of HCC tissue microarry.

Supplementary table 3. The univariate analysis and multivariate analysis in TCGA cohort.

**Supplementary figures**


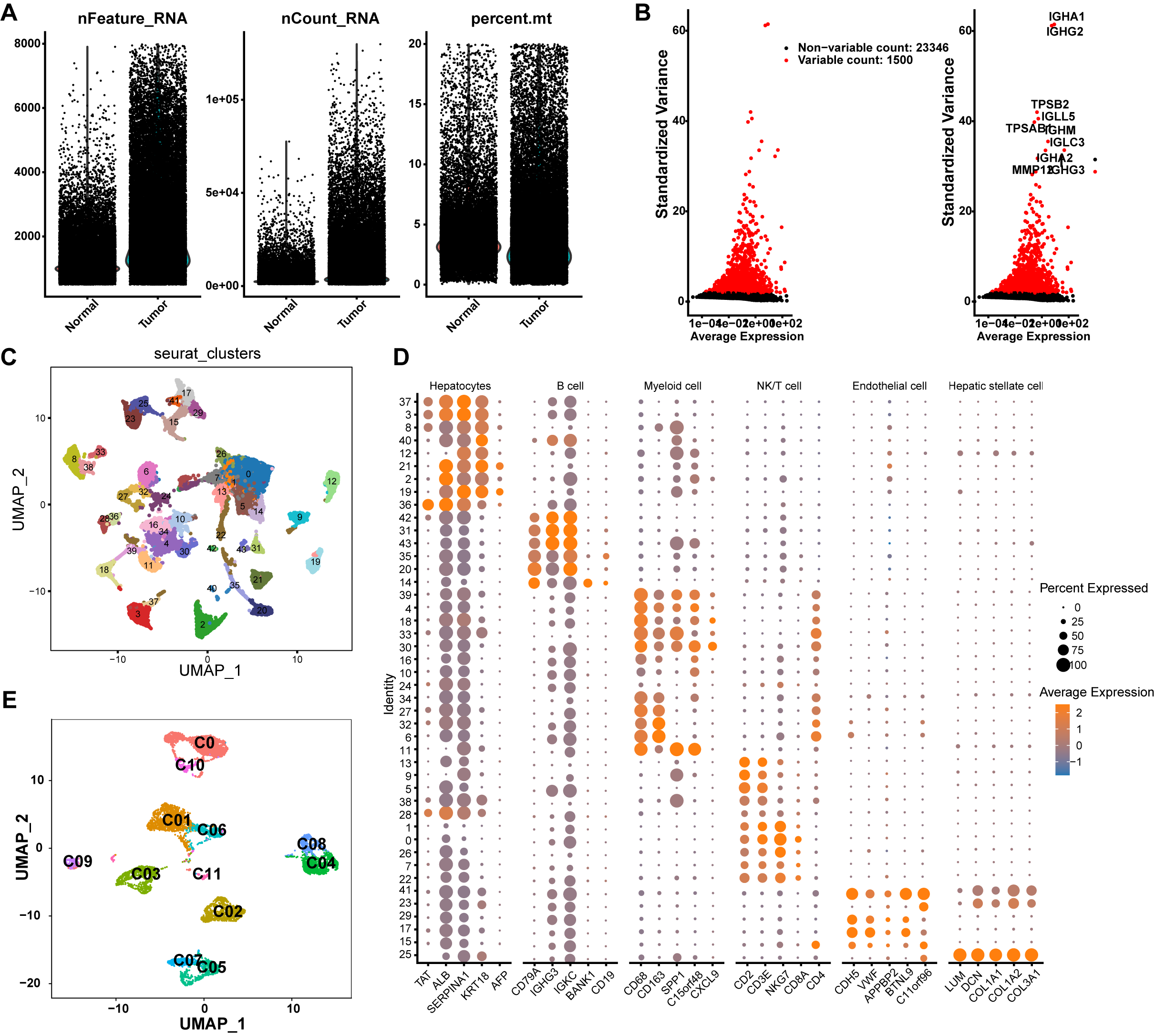


**Figure s1.Quality control process of single cell analysis in** **GSE149614. (A)** Quality control plots of cell samples. **(B)** 1500 variable genes and the top 10 variable genes across cell samples were identified. **(C)** UMAP plot showed [all](javascript:;) cells were classified into 44 clusters in GSE149614. **(D)**The expression of corresponding markers for diferent cells in all samples. **(E)** UMAP plot showed different clusters of hepatocytes.


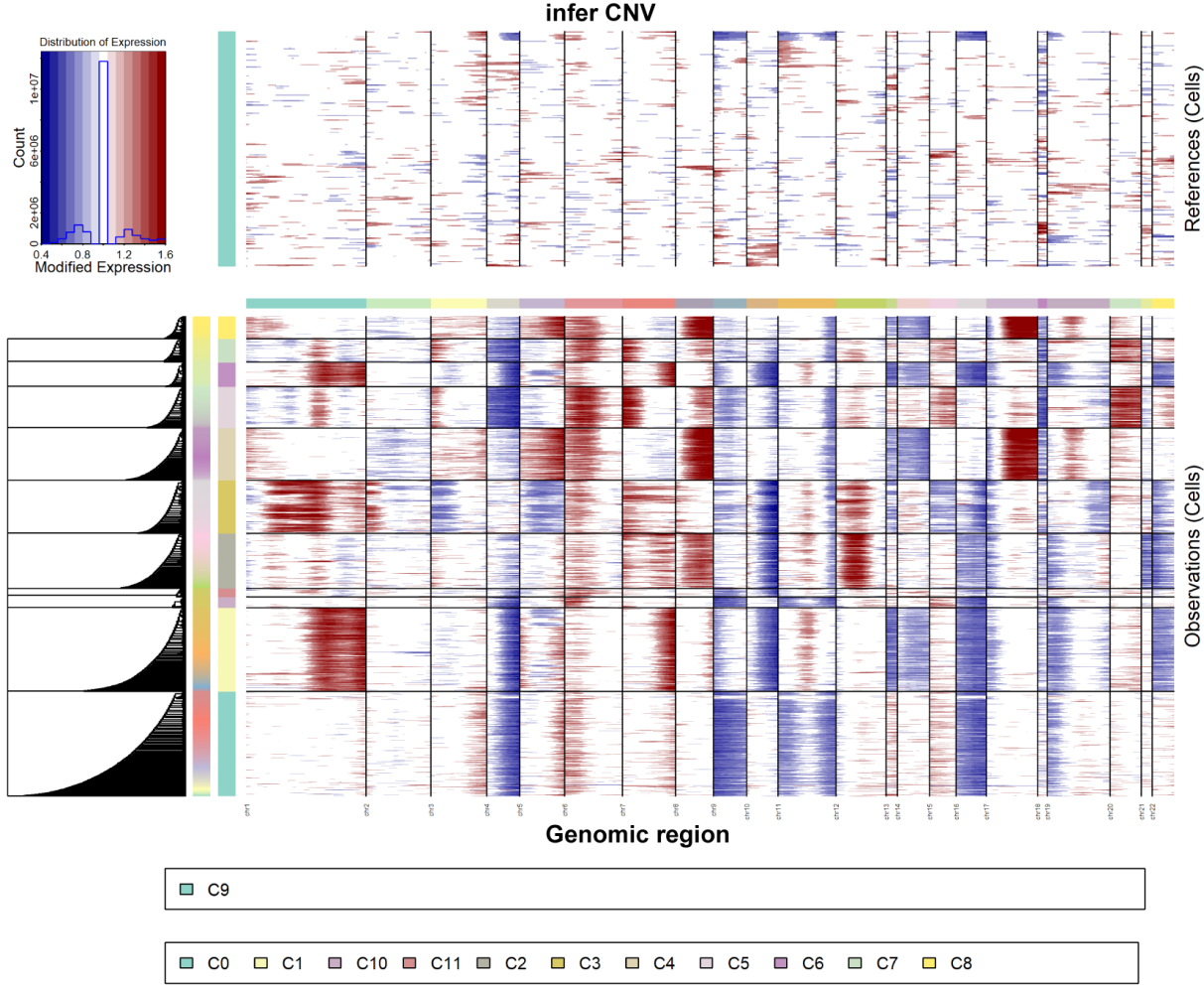


**Figure s2. The result of inferCNV analysis.**


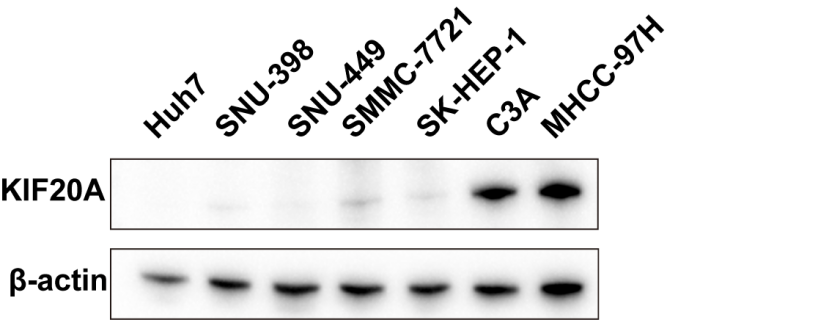


**Figure s3. The westernblot image showing the protein of KIF20A in different HCC cell lines.**


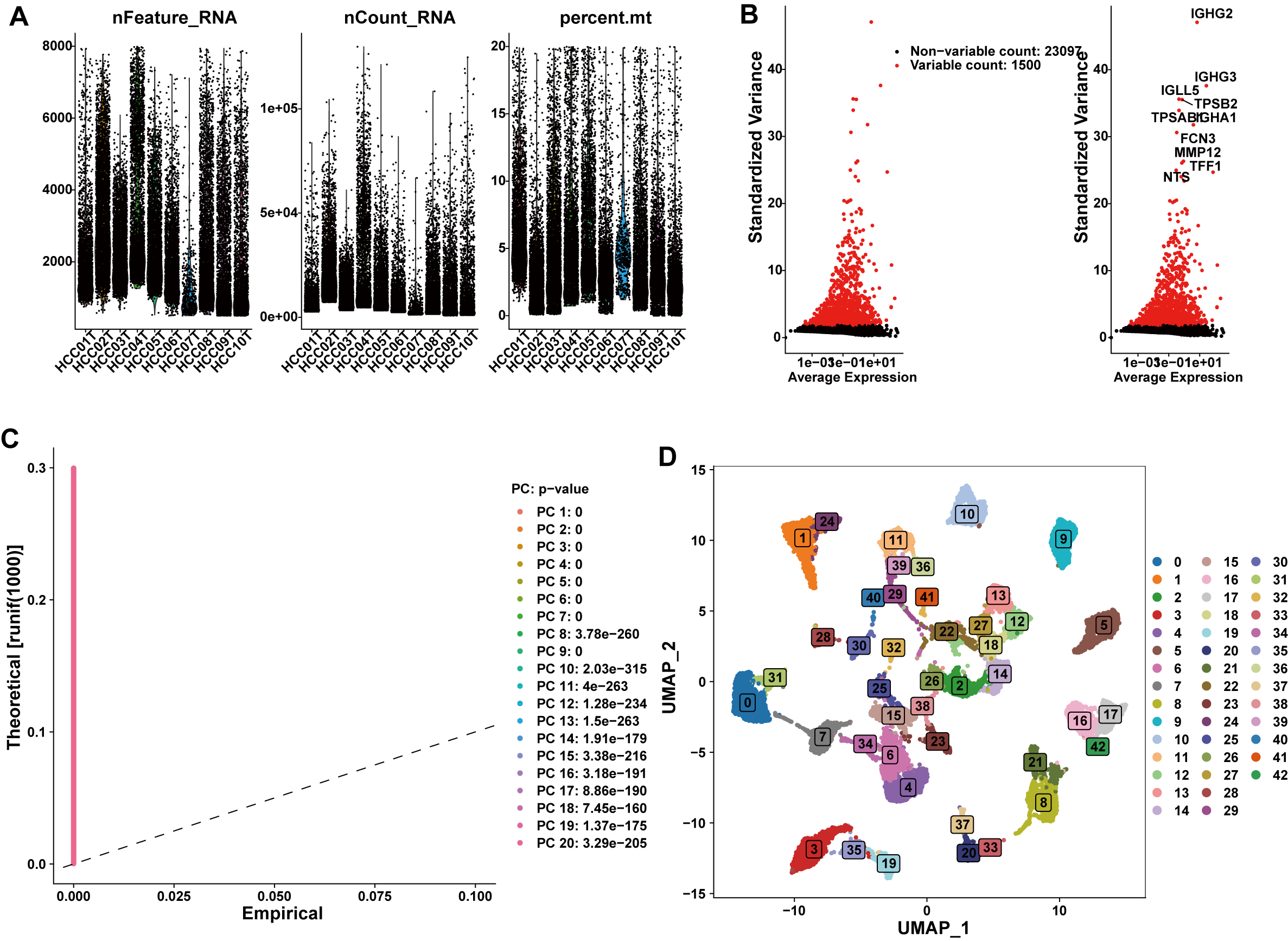


**Figure s4.Quality control process of single cell analysis in tumor samples of GSE149614. (A)** Quality control plots of tumor samples. **(B)** 1500 variable genes and the top 10 variable genes across cell samples were identified. **(C)** PCA was conducted to reduce the dimension of data sets.**(D)** [All](javascript:;) cells were classified into 43 clusters with the UMAP algorithm.


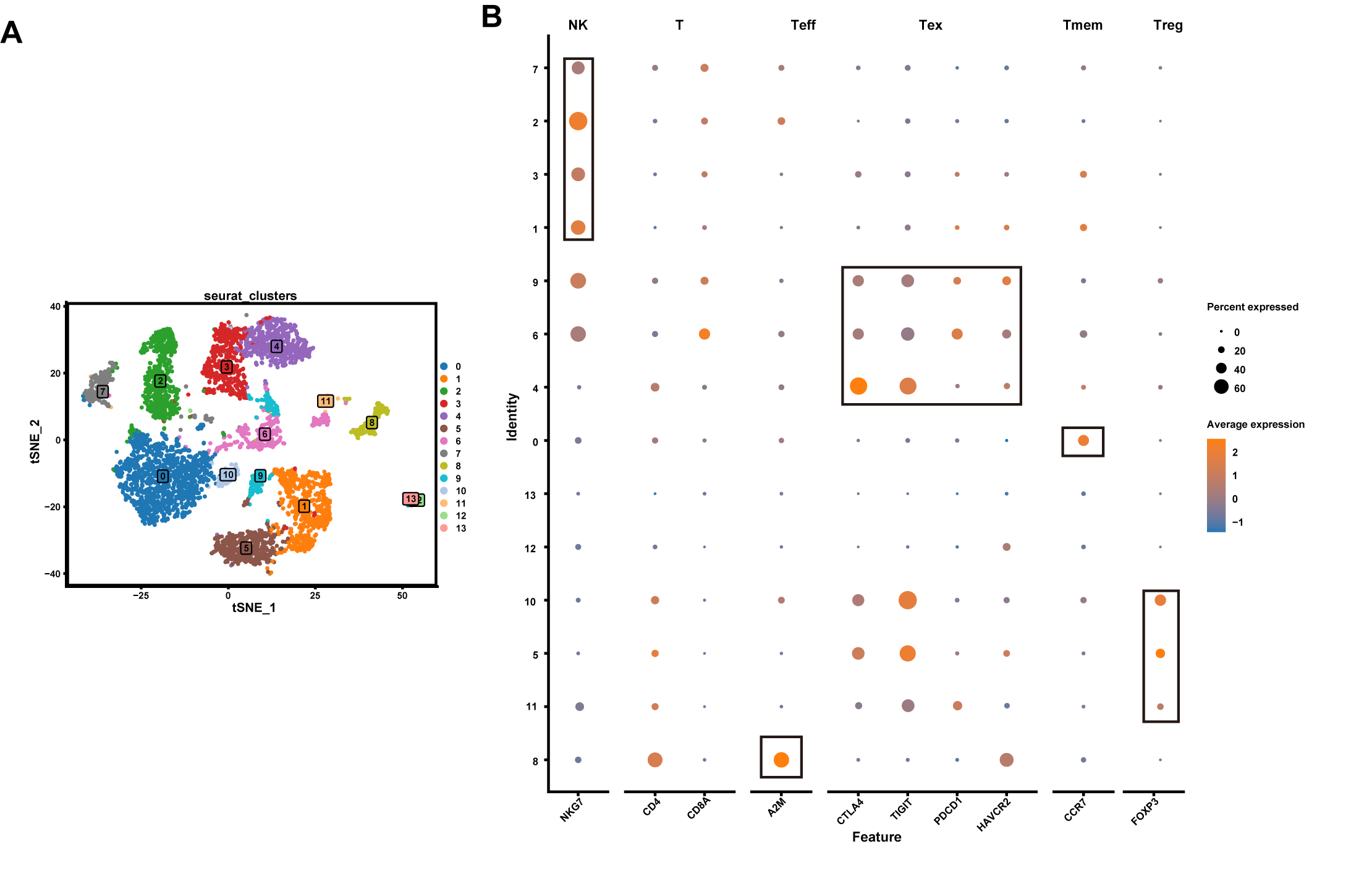
**Figure s5. Annotation of NK/T cell subsets.** **(A)** UMAP plot showing different clusters of NK/T cells in tumor samples. **(B)**The expression of corresponding markers for diferent cells.


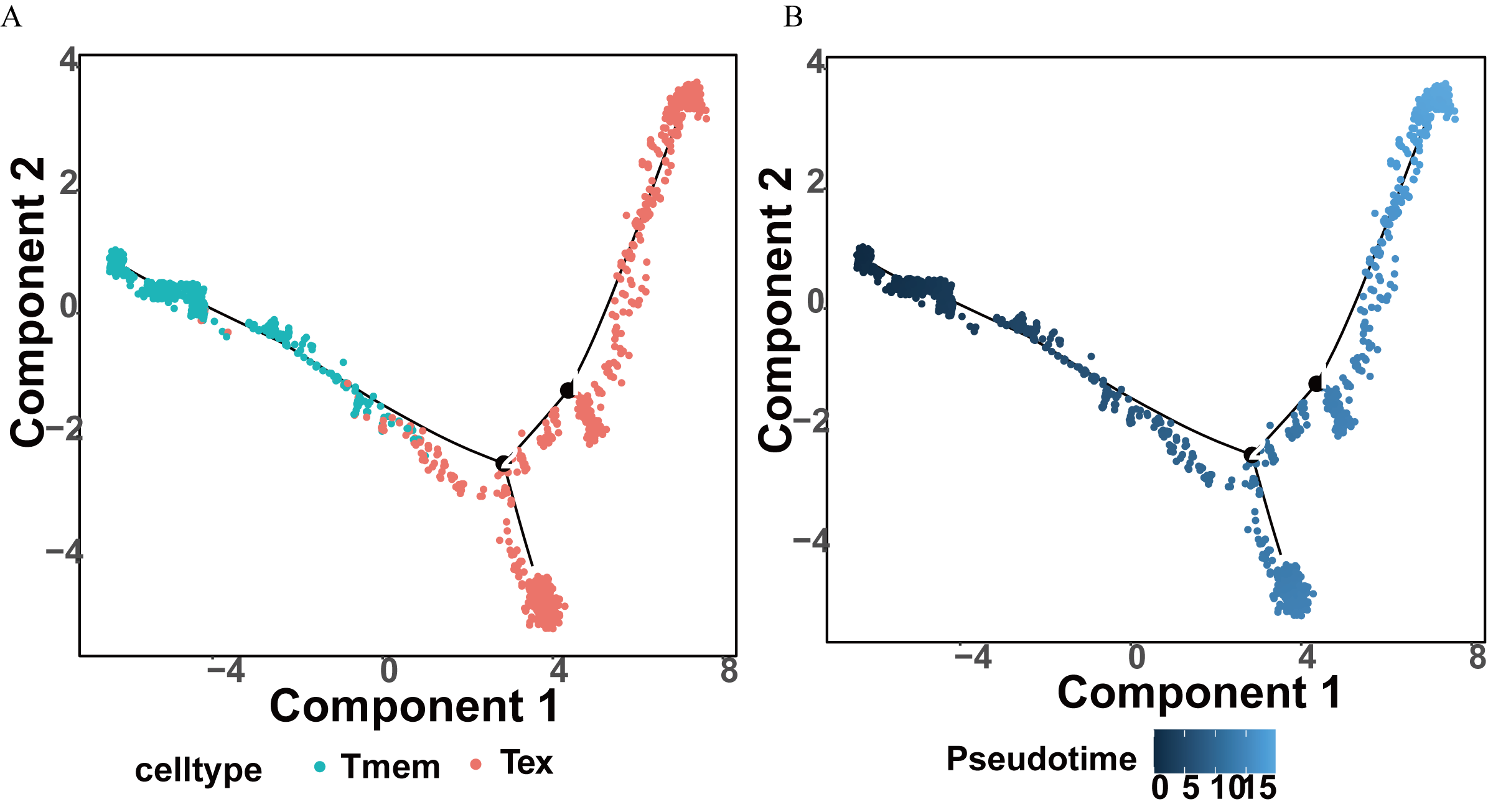
**Figure s6. The results of** **pseudotime analysis of Tmem and Tex cells.** (A) UMAP visualization demonstrated the pseudotime distribution pattern of Tmem and Tex cells. (B) UMAP visualized a pseudotime arrangement of Tmem and Tex cells.


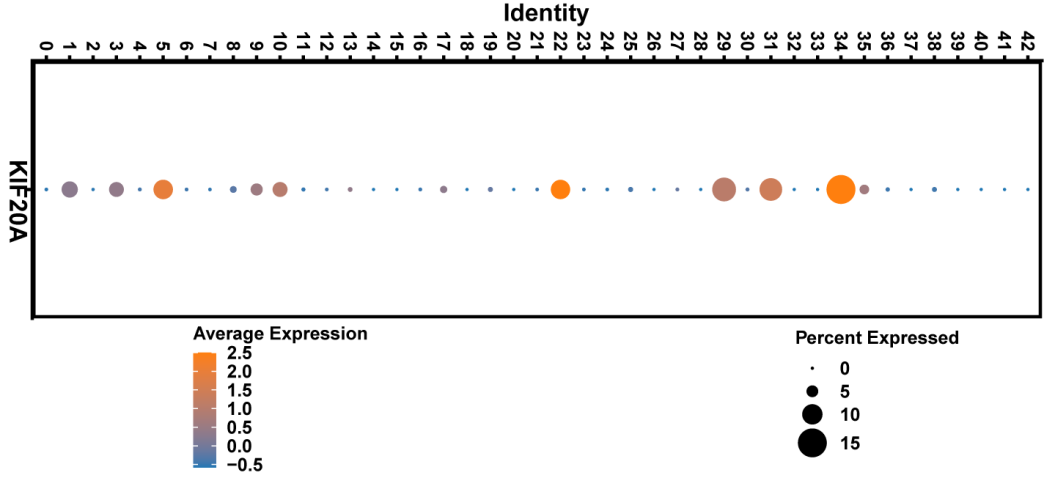


**Figure s7. The expression of KIF20A in each clusters in tumor samples.**


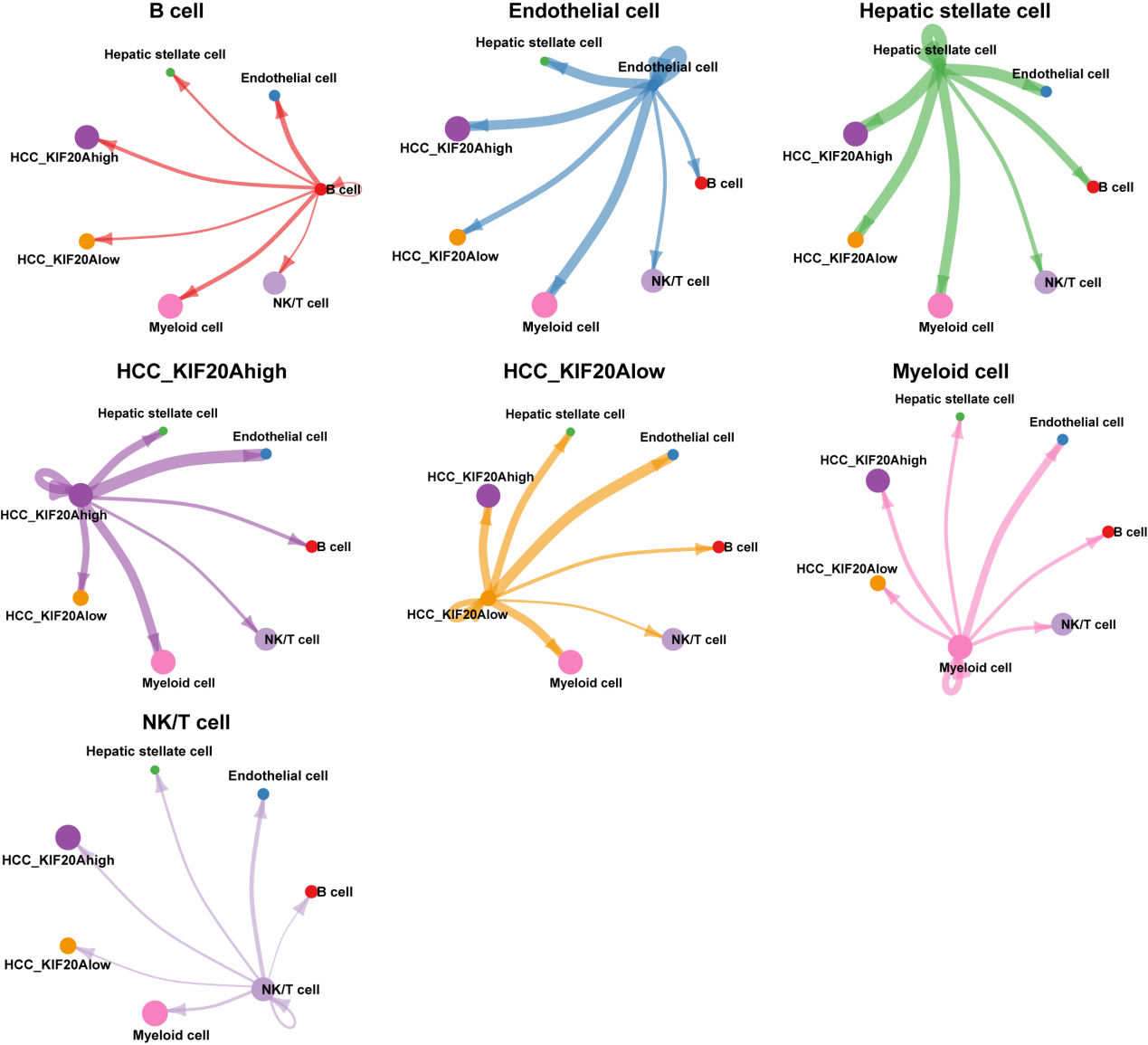
**Figure s8. The network plot showing the number of interactions between each cells to other cells in the TME.**


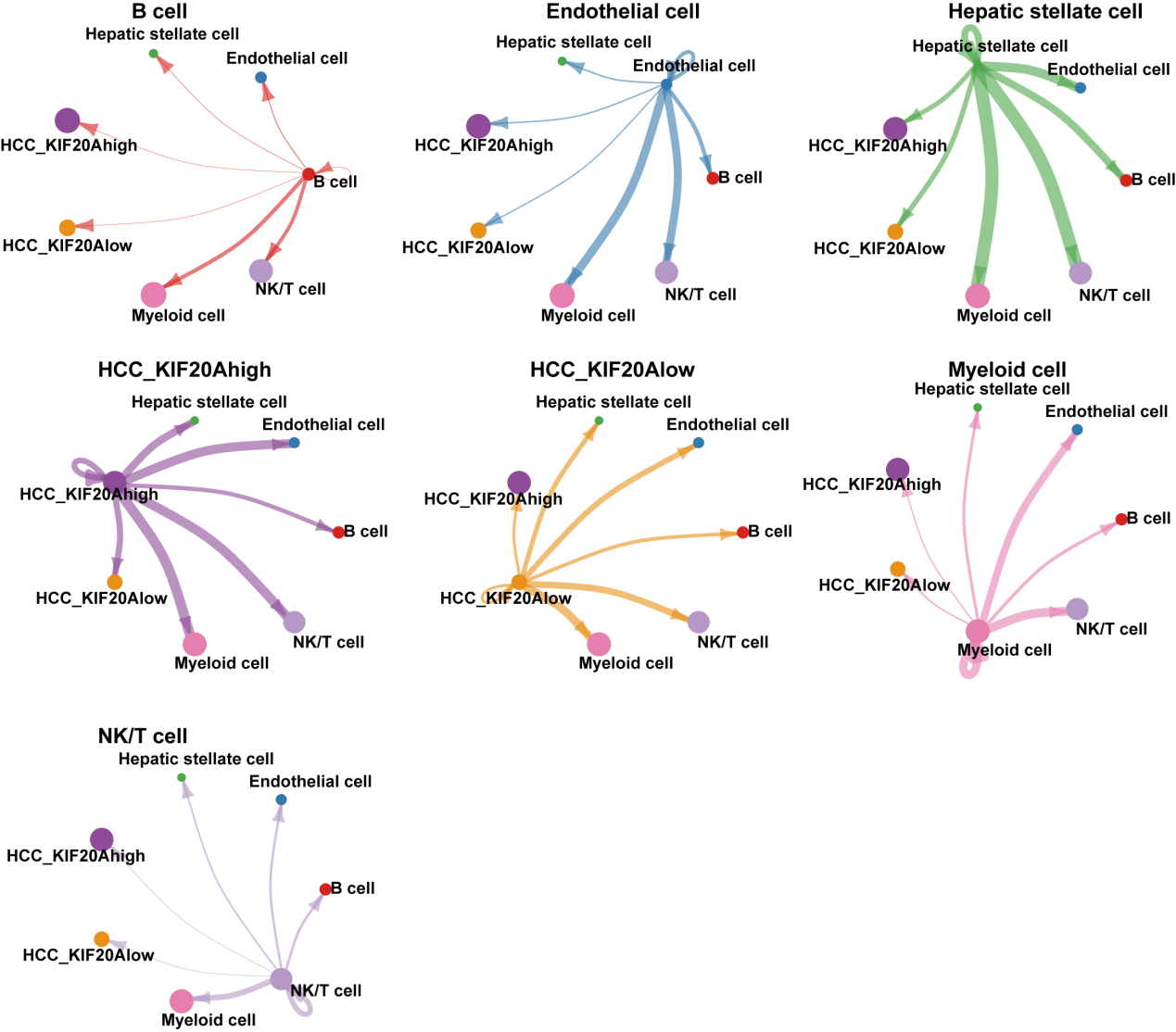
**Figure s9. The network plot showing the strength of interactions between each cells to other cells in the TME.**


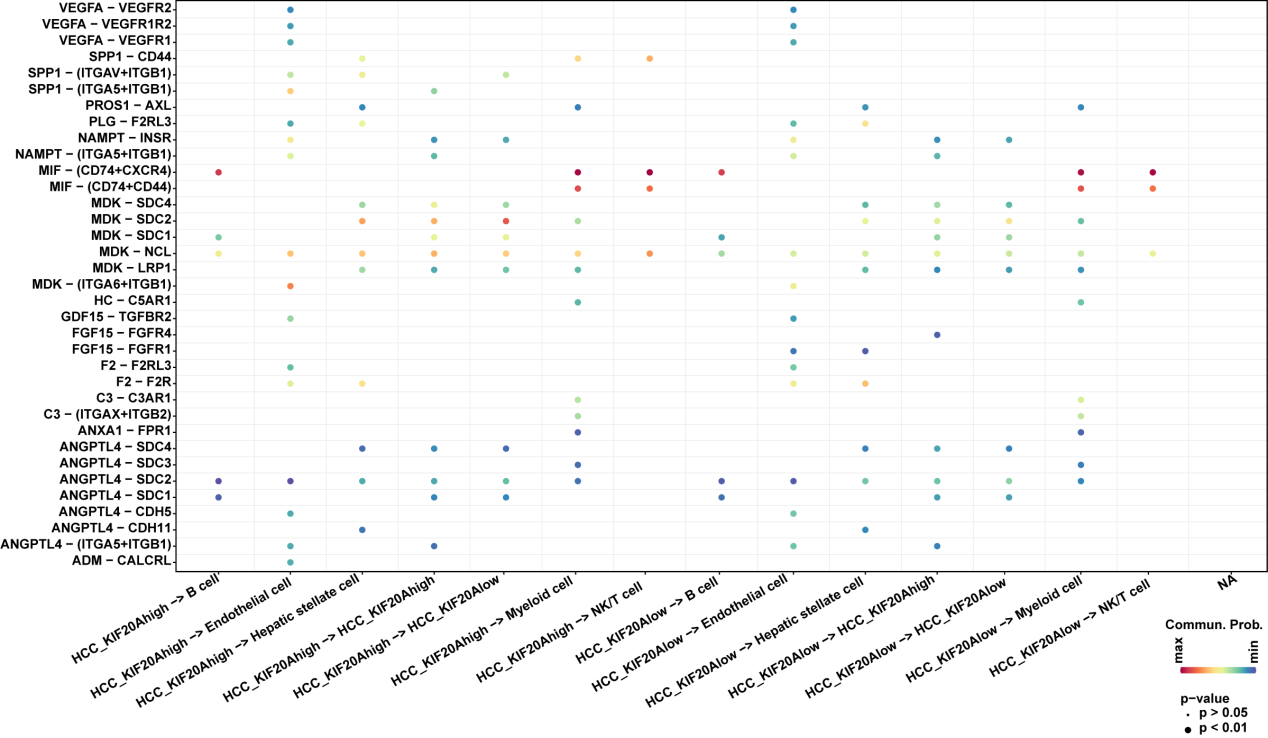
**Figure s10. Bubble plots showing the possible ligand-receptor pairs between KIF20Ahigh/low HCC cells and other cell subpopulations in the TME.**


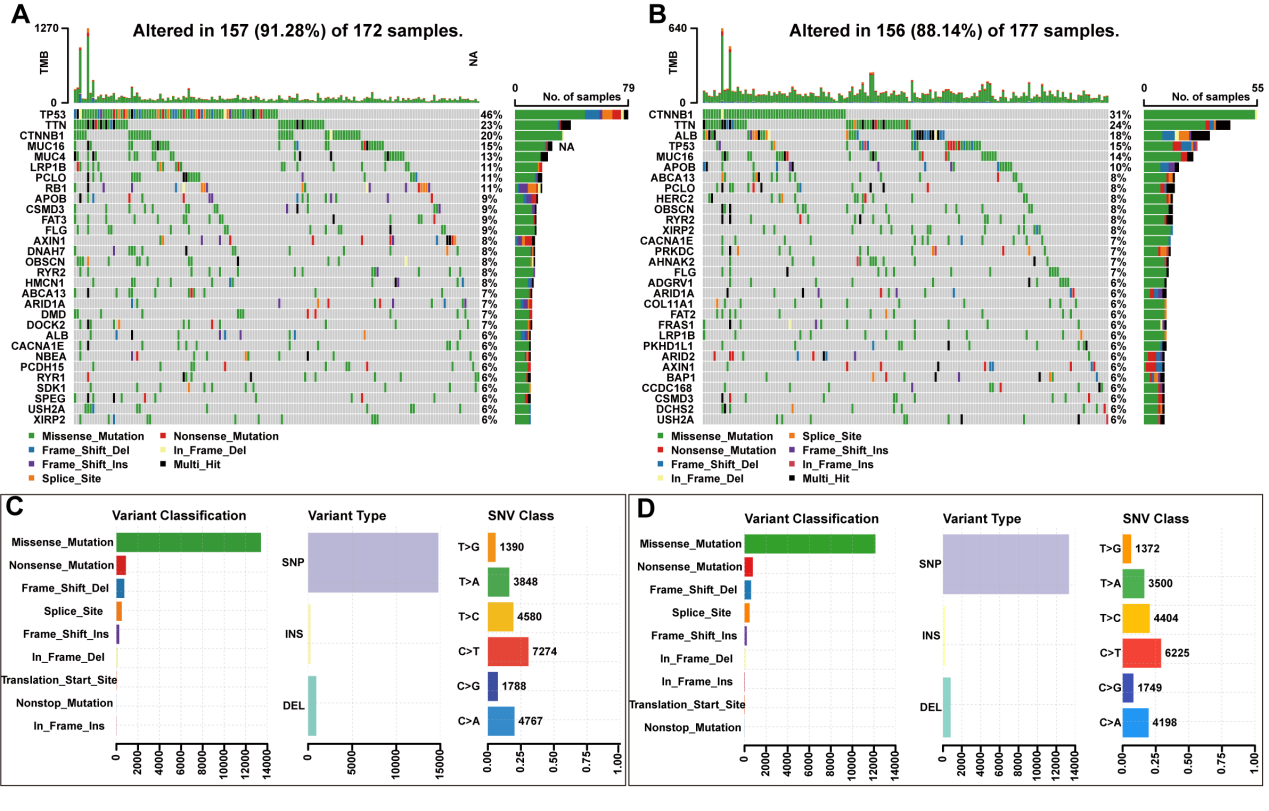
**Figure s11. Gene mutation analysis of different KID20A subgroups.** Mutated genes (rows, top 30) are ordered by mutation rate in the high-risk group **(A)** and low-risk group **(B)**. **(C)**Specific mutation types in KIF20A high expression samples. **(D)**Specific mutation types in KIF20A low expression samples.


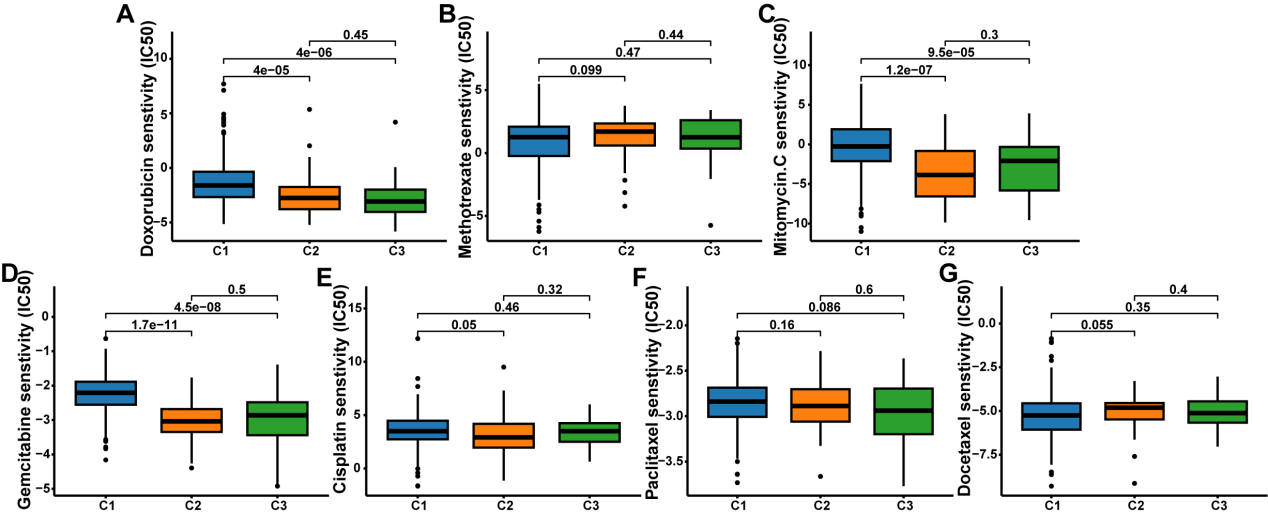
**Figure s12. Drug sensitivity of common chemotherapeutic agents.** The sensitivity of doxorubicin(A), methotrexate(B), mitomycin C(C), and gemcitabine(D), cisplatin (E), paclitaxel (F) and docetaxel (G) in different RPA clusters. Data are presented as mean ± SD. Statistical significance was calculated by one-way ANOVA with Tukey’s post hoc test. ns indicates no statistical difference, **P < 0.01.


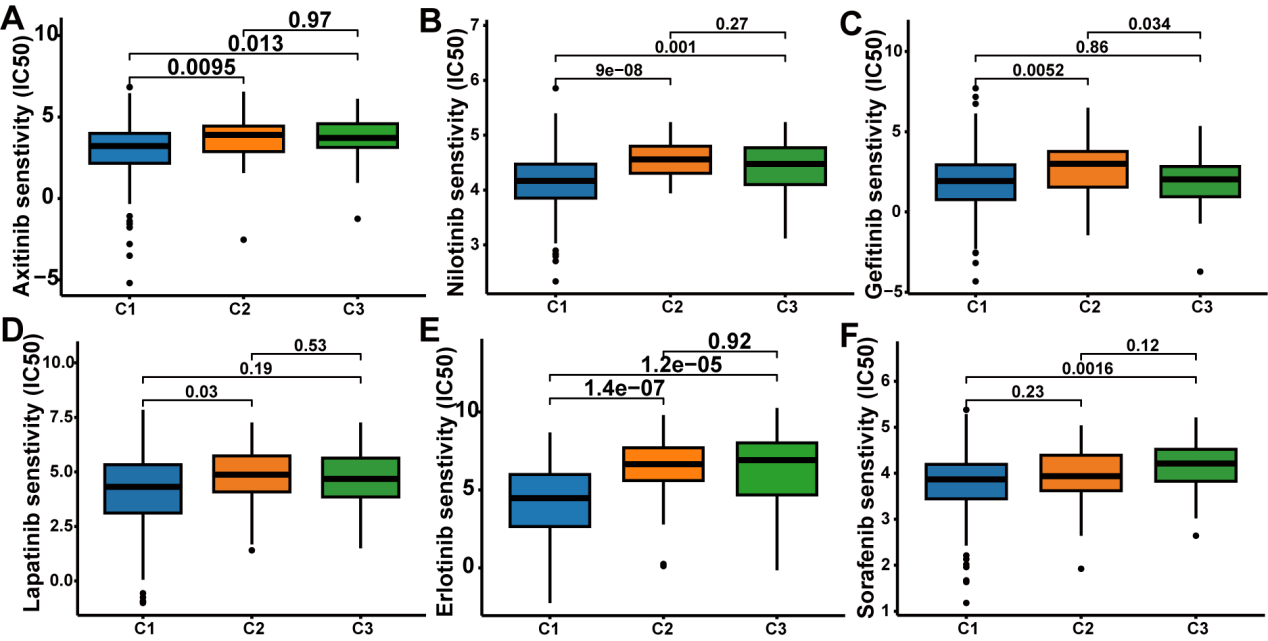
**Figure s13. Drug sensitivity of common targeted agents.** The sensitivity of axtinib(A), nilotinib(B), gefitinib(C), lapatinib(D), erlotinib(E), and sorafenib(F) in different RPA clusters. Data are presented as mean ± SD. Statistical significance was calculated by one-way ANOVA with Tukey’s post hoc test. ns indicates no statistical difference, **P < 0.01.
