# Supplementary material for: Bulk- and single cell-RNA sequencing reveal KIF20A as a key driver of hepatocellular carcinoma progression and immune evasion
Source: Front Immunol. 2024 Nov 1;15:1469827. doi: 10.3389/fimmu.2024.1469827 (PMC11563802; doi:10.3389/fimmu.2024.1469827)
Supplement: Supplementary file 4 [file Table3.docx]

| Characteristics | Total(N) | Univariate analysis | |  | Multivariate analysis | |
| --- | --- | --- | --- | --- | --- | --- |
|  |  | Hazard ratio (95% CI) | P value |  | Hazard ratio (95% CI) | P value |
| Age | 365 | 1.013 (0.999 - 1.027) | 0.073 |  | 1.010 (0.992 - 1.029) | 0.284 |
| Gender | 365 |  | 0.261 |  |  |  |
| MALE | 246 | Reference |  |  |  |  |
| FEMALE | 119 | 1.227 (0.861 - 1.749) | 0.257 |  |  |  |
| BMI | 332 | 1.000 (0.970 - 1.030) | 0.985 |  |  |  |
| AJCC_T | 362 |  | **< 0.001** |  |  |  |
| T2 | 91 | Reference |  |  | Reference |  |
| T1 | 180 | 0.696 (0.439 - 1.104) | 0.124 |  | 0.853 (0.465 - 1.566) | 0.608 |
| T3 | 78 | 1.822 (1.141 - 2.907) | **0.012** |  | 1.961 (1.102 - 3.488) | **0.022** |
| T4 | 13 | 3.687 (1.789 - 7.600) | **< 0.001** |  | 2.596 (0.896 - 7.520) | 0.079 |
| AJCC_N | 252 |  | 0.383 |  |  |  |
| N0 | 248 | Reference |  |  |  |  |
| N1 | 4 | 2.004 (0.491 - 8.181) | 0.333 |  |  |  |
| AJCC_M | 266 |  | 0.054 |  |  |  |
| M0 | 263 | Reference |  |  | Reference |  |
| M1 | 3 | 3.976 (1.250 - 12.648) | **0.019** |  | 2.406 (0.546 - 10.605) | 0.246 |
| Grade | 360 |  | 0.760 |  |  |  |
| G2 | 175 | Reference |  |  |  |  |
| G1 | 55 | 0.848 (0.501 - 1.437) | 0.541 |  |  |  |
| G3 | 118 | 1.046 (0.706 - 1.548) | 0.824 |  |  |  |
| G4 | 12 | 1.435 (0.574 - 3.589) | 0.439 |  |  |  |
| HBV | 365 |  | **0.001** |  |  |  |
| No | 287 | Reference |  |  | Reference |  |
| Yes | 78 | 0.467 (0.283 - 0.770) | **0.003** |  | 0.705 (0.383 - 1.297) | 0.261 |
| Alcohol | 365 |  | 0.901 |  |  |  |
| Yes | 121 | Reference |  |  |  |  |
| No | 244 | 0.977 (0.676 - 1.412) | 0.901 |  |  |  |
| recurrent | 365 |  | 0.111 |  |  |  |
| 1 | 179 | Reference |  |  |  |  |
| 0 | 186 | 0.749 (0.523 - 1.073) | 0.115 |  |  |  |
| AFP | 276 | 1.000 (1.000 - 1.000) | 0.432 |  |  |  |
| KIF20A | 365 | 1.148 (1.102 - 1.196) | **< 0.001** |  | 1.123 (1.069 - 1.181) | **< 0.001** |
